# 3-Substituted 1-Naphthamidomethyl-C-galactosyls Interact with Two Unique Sub-Sites for High-Affinity and High-Selectivity Inhibition of Galectin-3

**DOI:** 10.3390/molecules24244554

**Published:** 2019-12-12

**Authors:** Alexander Dahlqvist, Santanu Mandal, Kristoffer Peterson, Maria Håkansson, Derek T. Logan, Fredrik R. Zetterberg, Hakon Leffler, Ulf J. Nilsson

**Affiliations:** 1 Centre for Analysis and Synthesis, Department of Chemistry, Lund University, POB124, SE-22100 Lund, Sweden; alexander.dahlqvist@chem.lu.se (A.D.); santanuiiser@gmail.com (S.M.); kristoffer.peterson@redglead.com (K.P.); 2 SARomics Biostructures AB, Medicon Village, SE-223 63 Lund, Sweden; maria.hakansson@saromics.com (M.H.); derek.logan@biochemistry.lu.se (D.T.L.); 3 Biochemistry and Structural Biology, Center for Molecular Protein Science, Department of Chemistry, Lund University, POB124, SE-22100 Lund, Sweden; 4 Galecto Biotech AB, Sahlgrenska Science Park, Medicinaregatan 8 A, SE-413 46 Gothenburg, Sweden; FZ@Galecto.com; 5 Department of Laboratory Medicine, Section MIG, Lund University BMC-C1228b, Klinikgatan 28, 221 84 Lund, Sweden; hakon.leffler@med.lu.se

**Keywords:** galectin-3, inhibitor, selectivity, C-galactosyl, naphthamide

## Abstract

The galectins are a family of galactose-binding proteins playing key roles in inflammatory processes and cancer. However, they are structurally very closely related, and discovery of highly selective inhibitors is challenging. In this work, we report the design of novel inhibitors binding to a subsite unique to galectin-3, which confers both high selectivity and affinity towards galectin-3. Olefin cross metathesis between allyl β-C-galactopyranosyl and 1-vinylnaphthalenes or acylation of aminomethyl β-C-galactopyranosyl with 1-naphthoic acid derivatives gave C-galactopyranosyls carrying 1-naphthamide structural elements that interacted favorably with a galectin-3 unique subsite according to molecular modeling and X-ray structural analysis of two inhibitor-galectin-3 complexes. Affinities were down to sub-µM and selectivities over galectin-1, 2, 4 *N*-terminal domain, 4 C-terminal domain, 7, 8 *N*-terminal domain, 9 *N*-terminal domain, and 9 C-terminal domain were high. These results show that high affinity and selectivity for a single galectin can be achieved by targeting unique subsites, which holds promise for further development of small and selective galectin inhibitors.

## 1. Introduction

**Figure 1:**
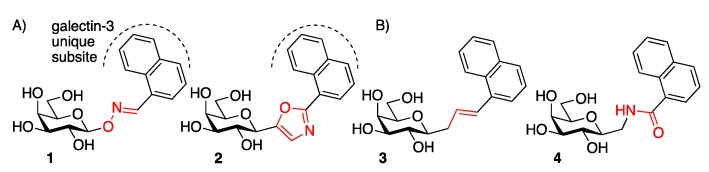
(**A**) Galectin-3 selective galactosyl aldoxime **1** and oxazole **2**. (**B**) The proposed bioisosteric alkene **3** and amide **4**.

Efforts towards the discovery of selective and drug-like inhibitors of the galectin family of proteins have escalated during the last decade as a consequence of an increasing awareness of the important roles of galectins in e.g., cell adhesion, tumor growth and metastasis, inflammation, and immune regulation [1,2]. Galectins may dimerize (e.g., galectin-1, 2, and 7), oligomerize (galectin-3), or have two ligand-binding sites within one polypeptide sequence (galectins-4, 6, 8, 9, and 12). These structural features enable crosslinking of endogenous glycoconjugates (e.g., glycoproteins) via oligosaccharides that carry the key ligand monosaccharide galactose [3,4,5]. Such cross-linking may in turn influence for example glycoprotein localization (on cell membrane or in intracellular vesicles) and cellular trafficking, which may influence glycoprotein function [6]. Endogenous oligosaccharide ligands and a majority of synthetic galactose-based inhibitors display at best partial selectivity among the galectins, which is why further development of inhibitors with enhanced selectivity is of significance. *O*-galactosyl aldoximes [7,8] and C1-galactopyranosyl oxazoles [9] carrying bicyclic aromatic *N*-substituents (Figure 1A) were earlier reported to be comparatively potent and highly selective galectin-3 inhibitors. A molecular modeling-based explanation was proposed in which the selectivity was due to anomeric aldoxime or oxazole substituents occupying and interacting favorably with a subsite unique to galectin-3 located perpendicular to the core galactoside subsite C conserved within galectins [4], with bicyclic aromatic substituents, such as the 1-naphthyl derivatives **1** and **2** (Figure 1A), being the most potent ones. Although these *O*-galactosyl aldoximes (**1**) and C1-galactosyl oxazoles (**2**) were selective and had improved affinity for galectin-3 over e.g., galactose, lactose, and *N*-acetyl-lactosamine, even further enhanced affinity is needed to improve pharmacological properties. Furthermore, the anomeric aldoxime functionality should preferably be replaced with functionalities less prone to undergo equilibrating reactions under physiological conditions, as an oxime ligand that exchanges with for example protein lysine side chains or other amino groups will likely possess altered galectin binding properties. Herein, we report on the synthesis and evaluation of anomeric aromatic amides and *trans*-alkenes as aldoxime and oxazole bioisosteres (Figure 1), leading to the discovery that amides possess galectin-3 affinities that reach the nanomolar range, while retaining the excellent selectivity earlier demonstrated for the known aldoximes and oxazoles. Furthermore, structural analysis of two anomeric 1-naphthyl amides in complex with galectin-3 confirmed that the naphthyl moiety occupied the site perpendicular to the conserved subsite C, while the amide N and O atoms were engaged in a hydrogen bond network with Q182-R186-R162 side chains, a buried water molecule, and the ligand galactose HO2, which is suggested to explain why the amide proved to be an ideal anomeric functional group over the aldoxime, oxazole, and *trans*-alkene.

## 2. Results and Discussion

**Scheme 1:**
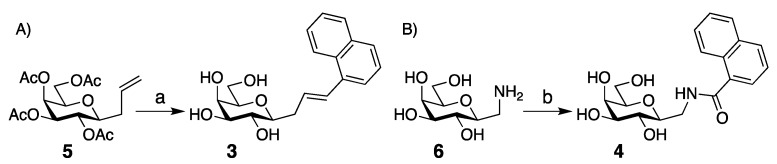
Synthesis of β-*C*-galactopyranosyls (**A**) alkene **3** and (**B**) amide **4**. Reagents and conditions: (a) ^i^ 1-vinylnaphthalene, Grubb’s catalyst 2nd generation, CH_2_Cl_2_, reflux, ^ii^ NaOMe, MeOH, 75% and (b) 1-naphthoyl chloride, Na_2_CO_3_, H_2_O, THF, 68%.

The alkene isostere **3** was synthesized via an olefin metathesis between allyl β-*C*-galactopyranosyl **5** [10] and 1-vinylnaphthalene, followed by deacetylation, and the amide **4** was synthesized by acylation of the aminomethyl β-*C*-galactopyranosyl **6** [11] (Scheme 1). The yields of the amides **4** and **7**–**11** were apparently low due to the presence of residual inseparable aluminum salts in C-galactosyl **5** resulting from its synthesis by LiAlH_4_ reduction of the preceding nitrile, as originally also reported by Coxon and co-workers for similar reactions [11].

**Scheme 2:**
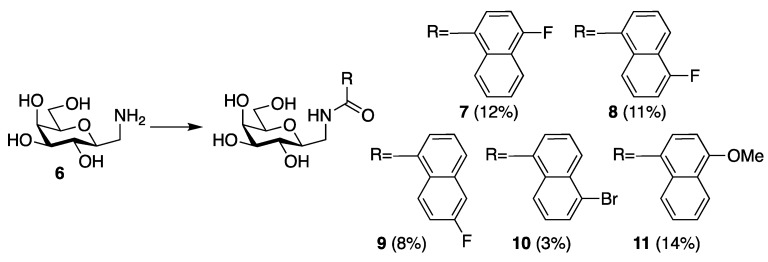
Synthesis of aromatic amidomethyl β-C-galactopyranosyls **7**–**11**. Reagents and conditions: Acyl chloride, Na_2_CO_3_, H_2_O, THF.

Evaluation of **3** and **4** as inhibitors of galectin-3 in a competitive fluorescence polarization assay [12,13], revealed that while the alkene **3** was a less efficient inhibitor than the parent aldoxime **1** and oxazole **2**, the amide **4** was significantly more potent (Table 1). Inspired by the discovery that amides were superior in binding to galectin-3, we synthesized a series of substituted bicyclic amides **7**–**11** by acylating **6** with different acyl chlorides following the same procedure as in the synthesis of **4** (Scheme 2). Substituent effects are very minor, as all substituted 1-naphthoyl derivatives **7**–**11** were all inhibitors in the low µM range (Table 1). The 6-fluoro derivative **8** was significantly weaker, while the 4-fluoro and 4-methoxy derivatives **7** and **11** were somewhat better than the other 1-naphthoyl derivatives.

**Scheme 3:**
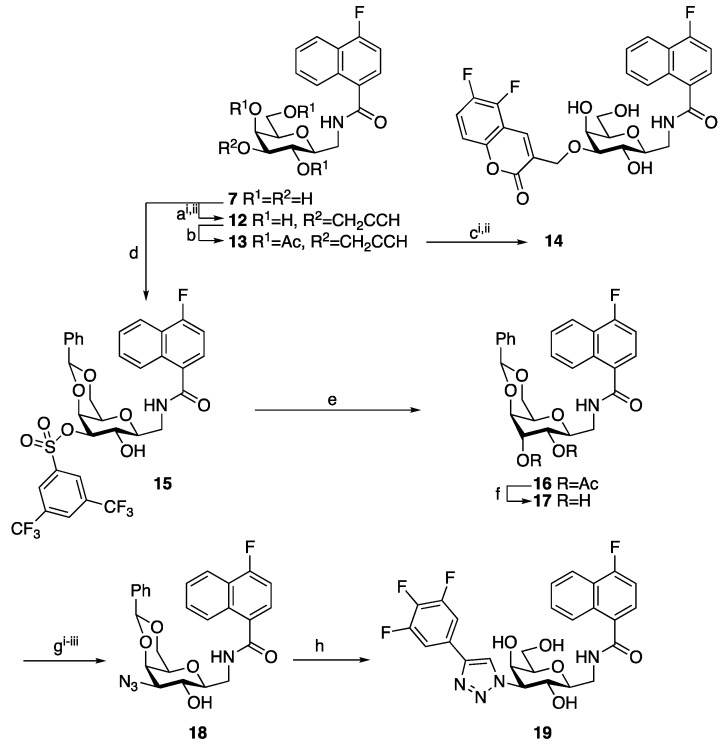
Synthesis of compounds **14** and **19**. Reagents and conditions: (a) ^i^ Bu_2_SnO, MeOH, ^ii^ propargyl bromide, 1,4-dioxane (79%); (b) Ac_2_O, pyridine (94%); (c) ^i^ 5,6-difluorosalicyldehyde, tosyl azide, Et_3_N, CuI, THF. ^ii^ NaOMe, MeOH (40%); (d) ^i^ benzaldehyde dimethyl acetal, p-toluenesulfonic acid, DMF. ^ii^ Bu_2_SnCl_2_, THF, PEMP, 3,5-bis-(trifluoromethyl)benzenesulfonyl chloride (52%); (e) ^i^ Ac_2_O, pyridine, ^ii^ CsOAc, DMSO, 90 °C, 3 days (35%); (f) NaOMe, MeOH (98%); (g) ^i^ dry CH_2_Cl_2_, pyridine, acetyl chloride, ^ii^ dry CH_2_Cl_2_, Tf_2_O, ^iii^ DMSO, NaN_3_, 60 °C, 8 h (15%); and (h) ^i^90% AcOH, 90 °C, 3 h, ^ii^ NaOMe, MeOH, ^iii^ DMF, 3,4,5-trifluorophenyl acetylene, CuI, Et_3_N, room temperature, 2 days (42%). Overall yields were 30% over 3 steps for the synthesis of **14** and 2% over 5 steps for the synthesis of **19** from the 4-fluoronaphthamide **7**.

With the 4-fluoro-1-naphthamide **7** being identified as one of two naphthamide ligands showing the highest affinity, the hypothesis that combining this C1-naphthamido moiety with known affinity-enhancing 5,6-difluorocoumarin- [15,16] and 3,4,5-trifluorophenyltriazolyl moieties [17,18] in the same manner as described for the aldoxime derivatives [8] to give the doubly derivatized C-galactosyls **14** and **19** (Scheme 3) appeared plausible. Stannylidene-mediated propargylation at HO3 of **7** gave the propargyl ether **12**. The remaining hydroxyl groups of **12** were acetylated to give **13** to be used as the alkyne source together with 5,6-difluorosalicylic aldehyde and tosyl azide for Cu(I)-mediated multicomponent coumarin synthesis [15,19] followed by de-acetylation to give the difluoro-coumarin **14**.

Access to the triazole group in **19** requires double inversion reactions at C3 of **7** to introduce an azido group for a Cu(I)-catalyzed cycloaddition reaction. Regioselective sulfonylation of **7** installed a bis-3,5-trifluoromethylbenzenethanolsulfonate at O3 (**15**), a leaving group recently demonstrated to possess optimal balance between reactivity and stability for inversions at galactose C3 [20]. Inversion of **15** with CsOAc in DMSO at 90 °C for 3 days gave the guloside **16**. De-acetylation of **16**, regioselective 2-*O*-acetylation, 3-*O*-triflation, and treatment with sodium azide gave the 3-azido derivative **18**. Acetate and benzylidene removal followed by Cu(I)-catalyzed cycloaddition [21,22] with 3,4,5-trifluorophenylacetylene gave **19** in 42% yield. Evaluation of **14** and **19** indeed revealed low to sub-µM affinities for galectin-3 (Table 2). While the coumarin **14** bound four-fold weaker to galectin-3 than **19**, it displayed a better selectivity over the other galectins evaluated, as **19** also inhibited galectin-4C (C-terminal domain) with low µM affinity.

**Figure 2:**
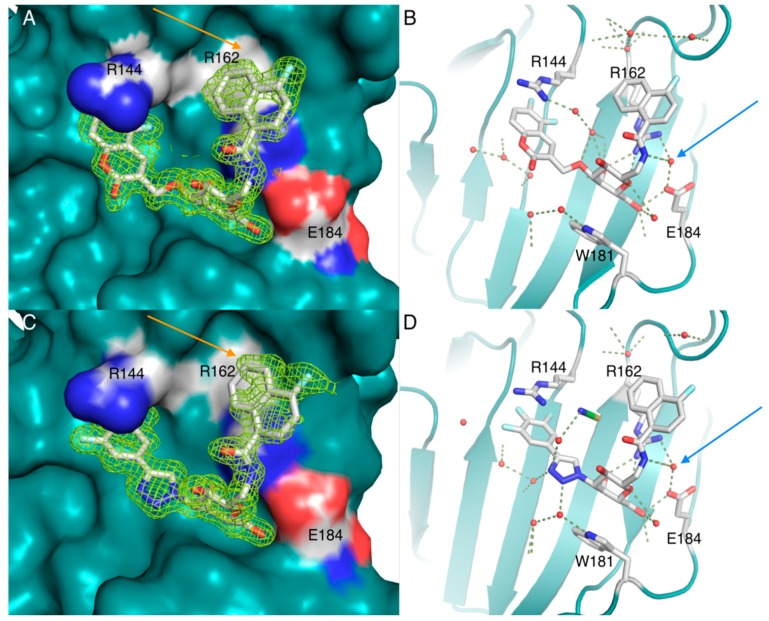
Electron densities of (**A**) **14** and (**C**) **19** in complex with galectin-3 (pdb id 6TF6 and 6TF7). Interactions of (**B**) **14** and (**D**) **19** in complex with galectin-3. The galectin-3-unique naphthyl-binding pocket above the β-face of the galactose residues is indicated with orange arrows and the water molecule hydrogen binding the amide NH and short-cutting the R162 and E184 salt-bridge analogously to ligand lactose HO3 is indicated with blue arrows.

In order to understand the structural basis for the affinities and selectivities of compounds **14** and **19**, the structures of their complexes with galectin-3 were determined with X-ray crystallography (Table 3 and Figure 2). Compounds **14** and **19** were soluble at 20 mg/mL, which allowed for co-crystallizations to be performed to generate crystals that diffracted down to 1.5 and 1.4 Å resolution, respectively, with **14** and **19**. The good solubility of 14 and 19 in the crystallization buffer is noteworthy as this suggests that both **14** and **19** can be dissolved in buffer at concentrations needed in cell or in vivo experiments involving selective galectin-3 inhibition. Both complexes revealed that the galactose ring of **14** and **19** bound the conserved galactose site of galectin-3 [25], which suggests that both **14** and **19** are specific for the glycan binding site (Figure 2). Furthermore, the naphthyl moieties of **14** and **19** indeed bound the pocket perpendicularly above the β-face of the galactoside site, which provides an explanation for the high galectin-3 selectivity of **14** and **19** as this pocket is unique to galectin-3. Furthermore, the naphthyl rings of **14** and **19** stack on top of and along the R162 side chain methylenes and guanidinium ion, which possibly accounts for some of the affinity enhancements of **14** and **19** via guanidinium-π/π-π and CH-π stacking interactions. The 4-fluoro atom of the naphthyls of **14** and **19** does not form any specific interactions with galectin-3, which may explain its marginal effects on affinity (cf. **4** and **7**). Given that compounds **4** and **7**–**11** binds with the same complex geometry as **14** and **19**, then any substituent influence on the naphthyl ring electron densities of **7**–**11** apparently has minor influence on the stacking interaction of the ligand naphthyl with R162 alkyl side chain atoms or guanidinium ion. The trifluorophenyl ring of **19** is placed in between the sidechain or R144 and the S3 β-strand, which is the same as recently described for thiodisaccharide- and galactose-based ligands [17,18,26]. The coumaryl moiety of **14** is similarly positioned between the R144 side chain and the S3 β-strand, which is different from that observed for earlier coumaryl-derivatized galactosides in which R144 is aligned along the protein surface and below the coumaryl moiety [15]. This is most probably caused by the interaction of the coumarin fluorine atoms of **14** forming multipolar C–F···H–N, C–F···C=O, and C-F···H–Cα interactions [27] (Figure 2B). The closest interactions are with the Cα of G238 (3.0 Å) and with ND2 of N160 (3.2 Å). In addition, longer fluorine-amide carbonyl interactions with residues R144, I145, and S237 are present (3.6–3.9 Å). Fluorine-amide carbonyl interactions have been observed for fluorophenyl-carrying ligands rings binding galectin-3 [17,18,28,29,30], but the magnitude of such interactions’ contribution to free energy of galectin-3 binding has been questioned [26].

**Figure 3:**
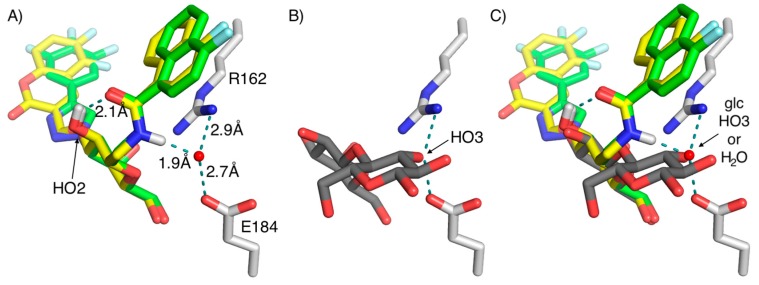
(**A**) The amide NH of **14** (yellow carbons) and **19** (green carbons) hydrogen binds a water molecule that short-cuts the R162-E184 salt-bridge in galectin-3. This hydrogen bonding network extends via the amide carbonyl to the ligand galactose HO2 (indicated with arrow). (**B**) The glucose HO3 (indicated with arrow) of lactose (grey carbons) replaces the water that short-cuts the R162-E184 salt-bridge in galectin-3 (from pdb id 3ZSJ [31]). (**C**) Overlay of **14**, **19**, and lactose showing that the lactose HO3 is positioned to short-cut the R162-E184 salt-bridge in the same way as the **14** and **19** amide NH hydrogen bonded water molecule.

Interestingly, the amide NH of both **14** and **19** formed a hydrogen bond to a water molecule short-cutting a salt-bridge between R162 and E184 (Figure 2B,D and Figure 3A). In natural ligands, for example lactose, this salt bridge is short-cut by the HO3 of the glucose subunit of lactose (Figure 3B). Hence, the amide NH of **14** and **19** hydrogen bonding to water mimics the similar hydrogen bonding pattern formed by, e.g., lactose or lacNAc-containing natural glycoconjugate ligands (Figure 3C). Furthermore, the hydrogen bond network with R162, E184, the water molecule, and **14** or **19** extends via the ligand amide oxygen to accept a hydrogen bond from the galactose HO2 (Figure 3A). This extended hydrogen bond network represents adds to the high **14**- and **19**-galectin-3 complex complementarity, which possibly contributes to the good affinity of **14** and **19** for galectin-3.

## 3. Conclusions

In conclusion, 1-naphthamido C-*β*-galactosyls have been synthesized and discovered to bind galectin-3 with high affinity and selectivity. The affinity enhancements and high selectivity stems from three factors. First, the 1-naphthamido moieties binding a pocket unique to galectin-3 and perpendicular to the core galactose binding site, in which it stacks onto the side chain and the guanidinium group of R162. Second, the trifluorophenyltriazolyl- and difluorocoumaryl-moieties at the galactose C3/O3 of **14** and **19**, respectively, are known to form affinity-enhancing stacking interactions with R144, as well as to confer selectivity for galectin-3. Finally, the amide NH-water mediated hydrogen bonds to R162 and E184 may not necessarily induce selectivity as is mimics a conserved hydrogen bonding motif involving the glucose or *N*-acetylglucosamine 3-OH of lactose or lacNAc as parts of endogenous ligands binding to several different galectins, but it apparently enhances affinity as seen when the amide **4** is compared to the oxime **1**, oxazole **2**, and alkene **3**.

## 4. Materials and Methods

### General

NMR spectra were recorded on a Bruker Avance II 400 MHz spectrometer (Fällanden, Switzerland) at ambient temperature. ^1^H-NMR spectra were assigned using 2D-methods (COSY). Chemical shifts are given in ppm downfield from the signal for Me_4_Si, with reference to residual CHCl_3_ or CD_2_HOD. High resolution mass spectrometry (HRMS) was determined by direct infusion on a Waters XEVO-G2 QTOF mass spectrometer (Waters, Milfor, MA, USA) using electrospray ionization (ESI). Reactions were monitored by TLC using aluminum-backed silica gel plates (Merck 60F_254,_ Darmstadt, Germany) and visualized using UV light and by charring with ethanolic H_2_SO_4_ (7%). Preparative chromatography was performed using silica gel (40–60 μm, 60 Å) columns. Solvents were dried by storing over activated M.S. Reagents were supplied by Sigma-Aldrich and used as is.

(*E*) 3-(β-d-galactopyranosyl)-1-(naphthalen-1-yl)-1-propene **3**:

To a solution of **5** (30 mg, 0.081 mmol) and Grubbs catalyst 2nd generation (3.4 mg, 0.0041 mmol) in CH_2_Cl_2_ (5 mL) was added 1-vinylaphthalene (62.5 mg, 0.405 mmol, 5 eq) and the resulting mixture was refluxed overnight. The solvent was evaporated and the residue dissolved in MeOH (5 mL), and NaOMe (1 M, 1 mL) was added. The mixture was stirred overnight, the pH was adjusted to 7 by addition of DOWEX 50W H^+^, and the solvent was evaporated. The residue was purified by flash chromatography (CH_2_Cl_2_:MeOH 9:1) to give **3** (20 mg, 75%). [α]_D_^20^ – 3.0 (*c* 1.32, MeOH). ^1^H NMR (CDCl_3_): δ 8.17 (d, *J* 8.0, 1H, Ph), 7.83 (d, *J* 8.0, 1H, Ph), 7.73 (d, *J* 8.1, 1H, Ph), 7.61 (d, *J* 7.1, 1H, Ph), 7.50–7.39 (m, 3H, Ph), 7.25 (d, *J* 15.5, 1H, CH), 6.44 (dt, *J* 15.6, 7.0, 1H, CH), 3.90 (d, *J* 3.3, 1H, H-4), 3.77 (dd, *J* 11.3, 6.8, 1H, H-6), 3.71 (dd, *J* 11.3, 5.4, 1H, H-6), 3.56 (t, *J* 9.3, 1H, H-2), 3.51 (t, *J* 6.1, 1H, H-5), 3.47 (dd, *J* 9.3, 3.3, 1H, H-3), 3.33 (obscured by CD_2_HOD, H-1), 2.88 (dd, *J* 14.9, 7.0, 1H, CH_2_), 2.57 (m, 1H, CH_2_). ^13^C NMR (CDCl_3_): δ 131.5, 130.0, 129.4, 128.3, 126.8, 126.6, 125.0, 124.5, 81.5, 80.3, 76.5, 72.3, 71.0, 62.9, 36.6. HRMS calcd for C_19_H_22_O_5_Na (M + Na)^+^: 353.1365: found: 353.1368.

Typical procedure for the synthesis of the compounds **4** and **7**–**11**: l-(4-Fluoro-naphth-1-amido)-2,6-anhydro-l-deoxy-d-glycero-l-manno-heptitol **7**:

To a solution of 1-C-aminomethyl-galactose **6** [11] (0.47 g, 2.43 mmol) in water (15 mL) was added a solution of 4-fluoro-1-naphthoyl chloride (0.76 g, 3.64 mmol) in THF (4 mL) followed by the addition of Na_2_CO_3_ (390 mg, 3.64 mmol). The reaction mixture was left overnight at room temperature. The solvent was evaporated and purified by flash chromatography (SiO_2_, CH_2_Cl_2_:MeOH 10:1) to afford pure **7** (0.102 g, 12%) as a colorless foam. The yield of **7** is apparently low due to the presence of residual inseparable aluminum salts in C-galactosyl **5** resulting from its synthesis by LiAlH_4_ reduction of the preceding nitrile, as originally also reported by Coxon and co-workers [11]. [α]_D_^25^ + 10.5 (*c* 1.1 in MeOH), ^1^H NMR (400 MHz, CD_3_OD) δ: 8.29–8.27 (m, 1H, ArH), 8.14–8.12 (m, 1H, ArH), 7.67–7.61 (m, 3H, ArH), 7.25 (t, 1H, *J* 8.0 Hz, ArH), 4.05 (t, 1H, *J* 2.8 Hz, H-1), 3.85 (d, 1H, *J* 2.4 Hz, H-4), 3.78 (dd, 1H, *J* 8.0, 7.5 Hz, H-6a), 3.66–3.48 (m, 5H, H-6b, H-2, H-3, CH_2_NH, H-5), 3.41–3.36 (m, 1H, CH_2_NH). ^13^C NMR (100 MHz, CD_3_OD) δ: 172.0, 133.3, 132.2, 129.1, 128.0, 127.1, 127.0, 126.7, 125.0, 121.4, 109.6, 80.5, 80.2, 76.08, 71.06, 70.2, 63.2, 42.5. HRMS calcd for C_18_H_20_FNO_6_Na (M + Na)^+^: 388.1172, found: 388.1176.

Compounds **4**, **8**–**11** were synthesized following the same procedure:

l-(Naphth-1-amido)-2,6-anhydro-l-deoxy-d-glycero-l-manno-heptitol **4**:

1-Naphthoyl chloride gave 30 mg (11%) of **4**. [α]_D_^25^ + 12.5 (*c* 1.3 in MeOH), ^1^H NMR (400 MHz, CD_3_OD) δ: 8.22 (dd, 1H, *J* 1.6, 0.4 Hz, ArH), 7.98 (d, 1H, *J* 8.4 Hz, ArH), 7.92 (dd, 1H, *J* 2.8, 2.0 Hz, ArH), 7.65 (dd, 1H, *J* 1.2 Hz, ArH), 7.58–7.49 (m, 3H, ArH), 4.07 (dd, 1H, *J* 2.4 Hz, H-1), 3.85 (d, 1H, *J* 2.4 Hz, H-4), 3.78 (dd, 1H, *J* 8.0, 7.6 Hz, H-6a), 3.65–3.48 (m, 5H, H-6b, H-2, H-3, CH_2_NH, H-5), 3.41–3.36 (m, 1H, CH_2_NH). ^13^C NMR (100 MHz, CD_3_OD) δ: 172.8, 135.8, 135.1, 131.5, 131.4, 129.4, 128.0, 127.4, 126.4, 126.4, 126.0, 80.5, 80.3, 76.1, 71.1, 70.2, 63.2, 42.5. HRMS calcd for C_18_H_21_NO_6_Na (M + Na)^+^: 370.1267, found: 370.1270.

l-(5-Fluoro-naphth-1-amido)-2,6-anhydro-l-deoxy-d-glycero-l-manno-heptitol **8**:

5-Fluoro-1-naphthoyl chloride gave 28 mg (11%) of **8**. [α]_D_^25^ + 9.7 (*c* 1.2 in MeOH), ^1^H NMR (400 MHz, CD_3_OD) δ: 8.76 (m, 1H, NH), 8.20 (d, 1H, *J* 8.4 Hz, ArH), 8.03 (d, 1H, *J* 8.4 Hz, ArH), 7.73 (t, 1H, *J* 1.2 Hz, ArH), 7.63 (t, 1H, *J* 6.8 Hz, ArH), 7.55–7.50 (m, 1H, ArH), 7.27–7.23 (m, 1H, ArH), 4.07–4.02 (m, 1H, H-1), 3.85 (d, 1H, *J* 2.8 Hz, H-4), 3.78 (dd, 1H, *J* 8.0, 7.6 Hz, H-6a), 3.66–3.48 (m, 5H, H-6b, H-2, H-4, CH_2_NH, H-5), 3.41–3.36 (m, 1H, CH_2_NH). ^13^C NMR (100 MHz, CD_3_OD) δ: 172.3, 135.9, 132.9, 128.0, 127.5, 126.6, 125.2, 125.0, 123.3, 122.7, 111.0, 80.5, 80.2, 76.1, 71.1, 70.2, 63.2, 42.5. HRMS calcd for C_18_H_20_FNO_6_Na (M + Na)^+^: 388.1172, found: 388.1173.

l-(6-Fluoro-naphth-1-amido)-2,6-anhydro-l-deoxy-d-glycero-l-manno-heptitol **9**:

6-Fluoro-1-naphthoyl chloride gave 57 mg (8%) of **9**. [α]_D_^25^ + 10.7 (*c* 1.4 in MeOH), ^1^H NMR (400 MHz, CD_3_OD) δ: 8.29 (t, 1H, *J* 5.6 Hz, ArH), 7.96 (d, 1H, *J* 8.0 Hz, ArH), 7.63–7.53 (m, 3H, ArH), 7.41–7.35 (m, 1H, ArH), 4.05 (t, 1H, *J* 2.8 Hz, H-1), 3.86 (t, 1H, *J* 0.8 Hz, H-4), 3.78 (dd, 1H, *J* 8.0, 7.6 Hz, H-6a), 3.66–3.48 (m, 5H, H-6b, H-2, H-5, CH_2_NH, H-3), 3.41–3.36 (m, 1H, CH_2_NH). ^13^C NMR (100 MHz, CD_3_OD) δ: 172.5, 136.2, 136.1, 135.9, 130.8, 129.5, 128.5, 127.2, 125.8, 118.1, 112.3, 80.5, 80.2, 76.1, 71.1, 70.2, 63.2, 42.5. HRMS calcd for C_18_H_20_FNO_6_Na (M + Na)^+^: 388.1172, found: 388.1174.

l-(5-Bromo-naphth-1-amido)-2,6-anhydro-l-deoxy-d-glycero-L-manno-heptitol **10**:

6-Bromo-1-naphthoyl chloride gave 18 mg (3%) of **10**. [α]_D_^25^ + 7.7 (*c* 1.4 in MeOH), ^1^H NMR (400 MHz, CD_3_OD) δ: 8.37 (d, 1H, *J* 8.4 Hz, ArH), 8.22 (d, 1H, *J* 8.4 Hz, ArH), 7.88 (dd, 1H, *J* 1.2, 0.8 Hz, ArH), 7.72–7.64 (m, 2H, ArH), 7.46 (dd, 1H, *J* 7.6, 7.2 Hz, ArH), 4.06 (t, 1H, *J* 2.4 Hz, H-1), 3.85 (d, 1H, *J* 2.4 Hz, H-4), 3.78 (t, 1H, *J* 8.0 Hz, H-6a), 3.66–3.48 (m, 5H, H-6b, H-2, H-3, CH_2_NH, H-5), 3.41–3.36 (m, 1H, CH_2_NH). ^13^C NMR (100 MHz, CD_3_OD) δ: 172.2, 136.7, 133.2, 132.8, 131.7, 130.0, 128.4, 127.6, 127.3, 126.7, 123.8, 80.5, 80.2, 76.1, 71.1, 70.2, 63.2, 42.5. HRMS calcd for C_18_H_20_BrNO_6_Na (M + Na)^+^: 448.0372, found: 448.0371.

l-(4-Methoxy-naphth-1-amido)-2,6-anhydro-l-deoxy-d-glycero-l-manno-heptitol **11**:

4-Methoxy-1-naphthoyl chloride gave 142 mg (14%) of **11**. [α]_D_^25^ + 11.4 (*c* 1.7 in MeOH), ^1^H NMR (400 MHz, CD_3_OD) δ: 8.56 (m, 1H, NH), 8.28 (2d, 2H, *J* 9.6 and 8.8 Hz, ArH), 7.64 (d, 1H, *J* 8.0 Hz, ArH), 7.58–7.47 (m, 2H, ArH), 6.92 (d, 1H, *J* 8.0 Hz, ArH), 4.03 (s, 3H, –OCH_3_), 4.01-3.99 (m, 1H, H-1), 3.84 (d, 1H, *J* 2.4 Hz, H-4), 3.78 (t, 1H, *J* 7.6 Hz, H-6a), 3.65–3.48 (m, 5H, H-6b, H-2, H-3, CH_2_NH, H-5), 3.39–3.34 (m, 1H, CH_2_NH). ^13^C NMR (100 MHz, CD_3_OD) δ: 173.0, 158.5, 132.7, 128.4, 127.9, 127.8, 126.8, 126.6, 126.3, 123.1, 103.7, 80.5, 80.3, 76.1, 71.1, 70.2, 63.2, 56.3, 42.5. HRMS calcd for C_19_H_23_NO_7_Na (M + Na)^+^: 400.1372, found: 400.1377.

l-(4-Fluoro-naphth-1-amido)-2,6-anhydro-4-*O*-propargyl-l-deoxy-d-glycero-l-manno-heptitol **12**:

Compound **6** (0.5 g, 1.36 mmol) was dissolved in dry MeOH (10 mL) and *n*-Bu_2_SnO (0.405 g, 1.63 mmol) was added. The mixture was refluxed at 70 °C for 3 h, where after the solvent was evaporated under reduced pressure. To the residue were added 1,4-dioxane (20 mL), propargyl bromide (365 μL, 4.08 mmol) and *n*-Bu_4_NBr (0.52 g, 1.63 mmol). The mixture was sonicated for 10 min and then refluxed overnight at 100 °C. Completion of reaction was checked by TLC (CH_2_Cl_2_:MeOH 6:1). The solvent was evaporated under reduced pressure and the sticky residue was dissolved in MeOH to obtain a slurry and purified by flash chromatography (SiO_2_, CH_2_Cl_2_: MeOH 17:1) to afford compound **12** (0.434 g, 79%). [α]_D_^25^ + 9.1 (*c* 0.54 in MeOH), ^1^H NMR (400 MHz, CD_3_OD) δ: 8.28 (dd, 1H, *J* 2.0, 1.6 Hz, ArH), 8.14 (dd, 1H, *J* 2.8, 2.0 Hz, ArH), 7.67–7.61 (m, 3H, ArH), 7.25 (t, 1H, *J* 8.0 Hz, ArH), 4.40 (dd, 2H, *J* 3.9, 2.4 Hz, CH_2_CCH), 4.10 (d, 1H, *J* 2.8 Hz, H-4), 4.05 (dd, 1H, *J* 13.6, 2.8 Hz, CH_2_NH), 3.78–3.51 (m, 6H, H-6a, H-5, H-6b, CH_2_NH, H-2, H-3), 3.46–3.41 (m, 1H, H-1), 2.89 (t, 1H, *J* 2.4 Hz, HCCCH_2_). ^13^C NMR (100 MHz, CD_3_OD) δ: 172.0, 133.2, 132.2, 129.1, 127.97, 127.95, 127.1, 126.7, 125.0, 121.4, 109.6, 83.0, 81.0, 80.3, 80.2, 76.1, 69.1, 67.9, 63.1, 57.7, 42.5. HRMS calcd for C_21_H_22_FNO_6_Na (M + Na)^+^: 426.1329, found: 426.1331.

l-(4-Fluoro-naphth-1-amido)-3,5,7-tri-*O*-acetyl-2,6-anhydro-4-*O*-propargyl-l-deoxy-d-glycero-l-manno-heptitol **13**:

Compound **12** (0.315 g, 0.78 mmol) was dissolved in pyridine (10 mL) and acetic anhydride (10 mL), stirred for 2 h, evaporated, and the residue was purified by column chromatography (SiO_2_, heptane:EtOAc 1:1) to give **13** (0.388 g, 94%). [α]_D_^25^ + 16.2 (*c* 1.1 in CHCl_3_), ^1^H NMR (400 MHz, CDCl_3_) δ: 8.38 (d, 1H, *J* 8.0 Hz, ArH), 8.15 (d, 1H, *J* 7.6, ArH), 7.65–7.57 (m, 3H, ArH), 7.14 (t, 1H, *J* 7.6 Hz, ArH), 6.37 (m, 1H, NH), 5.46 (dd, 1H, *J* 3.5, 1.0 Hz, H-4), 5.11 (t, 1H, *J* 10.0 Hz, H-3), 4.20 (d, 2H, *J* 2.4 Hz, OCH_2_CCH), 4.11 (dd, 2H, *J* 2.4, 1.6 Hz, H-6a, H-6b), 4.05–3.99 (m, 1H, CH_2_NH), 3.89–3.86 (m, 2H, H-3, H-5), 3.73–3.68 (m, 1H, H-1), 3.47–3.41 (m, 1H, OCH_2_CCH), 2.46 (t, 1H, *J* 2.4 Hz, HCCCH_2_), 2.16, 2.08, 1.96 (s, 9H, 3× COCH_3_). ^13^C NMR (100 MHz, CDCl_3_) δ: 170.6, 170.5, 170.4, 168.9, 132.1, 130.7, 128.3, 127.0, 125.63, 125.57, 125.5, 124.1, 121.0, 108.6, 79.2, 76.9, 76.7, 75.2, 74.8, 68.1, 66.3, 62.3, 56.6, 40.5, 21.1, 20.9, 20.8. HRMS calcd for C_27_H_28_FNO_9_Na (M + Na)^+^: 552.1646, found: 552.1650.

l-(4-Fluoro-naphth-1-amido)-2,6-anhydro-4-*O*-(5,6-difluoro-2H-1-benzopyran-2-on-3-yl)-l-deoxy-d-glycero-l-manno-heptitol **14**:

Compound **13** (0.117 g, 0.22 mmol) was dissolved in dry THF (10 mL) to which TsN_3_ (0.052 g, 0.264 mmol), 5,6-difluorosalicylaldehyde (0.042 g, 0.264 mmol), and CuI (0.0042 g, 0.022 mmol) was added. After 5 min, Et_3_N (62 μL, 0.44 mmol) was added to it resulting in the change of the color of the solution. The reaction mixture was left overnight and TLC was checked. After consumption of the starting material, the solution was evaporated and diluted with CH_2_Cl_2_. Work up was done with NH_4_Cl solution. The organic layer collected was evaporated and directly proceeded to the next step. The compound was dissolved in MeOH (5 mL) and freshly prepared NaOMe (0.5 M in MeOH, 100 mL) was added to it and left for 2 days. After complete conversion of the starting material, the solution was neutralized with DOWEX 50W H+ resin, filtered, evaporated and purified by flash chromatography (SiO_2_, CH_2_Cl_2_: MeOH 20:1) to afford colorless pure compound **14** (0.050 g, 40%). [α]_D_^25^ + 3.7 (*c* 0.33 in MeOH), ^1^H NMR (400 MHz, CD_3_OD) δ: 8.37 (s, 1H, ArH), 8.30–8.27 (m, 1H, ArH), 8.13–8.10 (m, 1H, ArH), 7.67–7.60 (m, 3H, ArH), 7.52–7.45 (m, 1H, ArH), 7.25–7.17 (m, 2H, ArH), 4.74 (dd, 1H, *J* 1.6 Hz, CH_2_Ph), 4.61 (t, 1H, *J* 1.2 Hz, CH_2_Ph), 4.18 (d, 1H, *J* 2.4 Hz, H-3), 4.08 (dd, 1H, *J* 2.8, 2.4 Hz, CH_2_NH), 3.83–3.77 (m, 2H, H-6a, H-5), 3.70–3.55 (m, 3H, H-6b, CH_2_NH, H-4), 3.52–3.41 (m, 2H, H-2, H-1). ^13^C NMR (100 MHz, CD_3_OD) δ: 172.0, 161.1, 159.7 133.2, 132.2, 132.0, 129.1, 128.0, 127.14, 127.05, 126.7, 125.0, 121.41, 121.35, 120.5, 120.3, 113.4, 109.6, 109.4, 84.7, 80.3, 80.2, 69.2, 67.6, 66.8, 63.2, 42.5. HRMS calcd for C_28_H_24_F_3_NO_8_Na (M + Na)^+^: 582.1352, found: 582.1350.

l-(4-Fluoro-naphth-1-amido)-2,6-anhydro-5,7-*O*-benzylidene-4-*O*-[3,5-bis-(trifluoromethyl)-benzenesulfonyl]-l-deoxy-d-glycero-l-manno-heptitol **15**:

Compound **7** (0.332 g, 0.908 mmol) was dissolved in DMF (10 mL) under nitrogen, followed by adding benzaldehyde dimethyl acetal (204 μL, 1.36 mmol) and a catalytic amount of *p*-toluene sulfonic acid. The reaction mixture was stirred for 2.5 h at room temperature and reaction completion was confirmed by TLC. Then the solution was diluted with ethyl acetate (20 mL) and washed successively with H_2_O (2 × 10 mL) and brine (20 mL). The organic layer was separated, dried (Na_2_SO_4_) and evaporated as pale yellow syrup, which was pure enough to proceed to the next step. The compound (260 mg, 0.57 mmol) was dissolved in THF (30 mL) to which Bu_2_SnCl_2_ (17 mg, 0.057 mmol) was added under nitrogen atmosphere and the mixture was stirred at room temperature for 15 min. A mixture of 1,1,2,2,6-pentamethylpiperidine (260 μL, 1.14 mmol) and 3,5-bis-(trifluoromethyl)benzenesulfonyl chloride (197 mg, 0.63 mmol) in THF, was added to it. The mixture was stirred overnight at room temperature until the starting material was completely consumed (TLC). The solution was quenched by NH_4_Cl solution and the solvents were evaporated to give the crude product. The solution was then diluted with ethyl acetate (20 mL) and washed successively with H_2_O (2 × 10 mL) and brine (10 mL). The organic layer was separated, dried (Na_2_SO_4_) and evaporated in vacuo. The crude product was purified by flash chromatography (SiO_2_, heptane:EtOAc 1:1) to afford pure **15** (216 mg, 52%) as white foam. [α]_D_^25^ + 6.2 (*c* 0.56 in CHCl_3_), ^1^H NMR (400 MHz, CDCl_3_) δ: 8.45 (s, 2H, ArH), 8.24 (d, 1H, *J* 8.0 Hz, ArH), 8.10–8.07 (m, 2H, ArH), 7.54 (t, 1H, *J* 7.2 Hz, ArH), 7.45–7.33 (m, 5H, ArH), 7.24 (dd, 2H, *J* 8.0, 7.2 Hz, ArH), 6.67–6.61 (m, 2H, ArH), 5.58 (s, 1H, CHPh), 4.78 (dd, 1H, *J* 3.2 Hz, H-3), 4.56 (d, 1H, *J* 3.2 Hz, H-4), 4.36–4.25 (m, 3H, CH_2_NH, H-2, H-6a), 4.08–4.02 (m, 2H, H-5, H-6b), 3.58 (s, 2H, OH), 3.46 (d, 1H, *J* 9.6 Hz, H-1), 3.33–3.27 (m, 1H, CH_2_NH). ^13^C NMR (100 MHz, CDCl_3_) δ: 171.4, 161.6, 159.1, 139.6, 137.4, 132.9, 132.6, 131.82, 131.8, 129.2, 128.7, 128.4, 127.1, 126.4, 126.3, 126.0, 125.1, 124.1, 123.88, 123.85, 121.1, 121.0, 120.9, 108.6, 108.4, 100.5, 83.0, 79.5, 75.3, 69.6, 69.2, 64.5, 40.2. HRMS calcd for C_33_H_26_F_7_NO_8_SNa (M + Na)^+^: 752.1165, found: 752.1170.

l-(4-Fluoro-naphth-1-amido)-3,4-di-*O*-acetyl-2,6-anhydro-5,7-*O*-benzylidene-l-deoxy-d-glycero-l-ido-heptitol **16**:

To a solution of compound **15** (216 mg, 0.29 mmol) in dry pyridine (10 mL) was added Ac_2_O (10 mL) and stirring was continued for 2 h at room temperature until the starting material was completely consumed according to TLC analysis. The solvents were evaporated in vacuo and the residue (198 mg, 0.256 mmol) was dissolved in DMSO (7 mL) followed by addition of CsOAc (147 mg, 0.769 mmol). The reaction mixture was stirred at 90 °C for 3 days until the starting material was completely consumed as confirmed by TLC. Then the reaction mixture was diluted by water (10 mL) and worked up with EtOAc (3 × 10 mL). The organic layer was separated and dried over Na_2_SO_4_ and evaporated. It was purified by flash chromatography (SiO_2_, hexane-EtOAc 1:1) to afford pure compound **16** (41 mg, 35%). [α]_D_^25^ +5.9 (*c* 1.1 in CHCl_3_), ^1^H NMR (400 MHz, CDCl_3_) δ: 8.36–8.34 (m, 1H, ArH), 8.12–8.09 (m, 1H, ArH), 7.58–7.52 (m, 3H, ArH), 7.40–7.32 (m, 3H, ArH), 7.27–7.23 (m, 2H, ArH), 6.93 (dd, 1H, *J* 8.0, 7.6 Hz, ArH), 6.56 (t, 1H, *J* 6.0 Hz, NH), 5.53 (m, 2H, CHPh, H-3), 5.29 (dd, 1H, *J* 10.4, 3.2 Hz, H-2), 4.28 (dd, 1H, *J* 12.6, 1.7 Hz, H-6a), 4.13–4.02 (m, 2H, H-4, H-1), 4.05 (dd, 1H, *J* 12.6, 1.8 Hz, H-6b), 3.81–3.78 (m, 2H, CH_2_NH), 3.73 (s, 1H, H-5), 2.18, 2.09 (s, 6H, 2× COCH_3_). ^13^C NMR (100 MHz, CDCl_3_) δ: 169.8, 169.6, 169.1, 161.3, 158.7, 137.3, 129.3, 128.4, 128.2, 126.9, 126.1, 125.73, 125.69, 125.67, 125.6, 120.8, 120.7, 108.6, 108.4, 101.1, 74.5, 72.1, 69.4, 67.9, 67.0, 65.9, 40.0, 21.0, 20.9. HRMS calcd for C_29_H_28_FNO_8_Na (M + Na)^+^: 560.1697, found: 560.1699.

l-(4-Fluoro-naphth-1-amido)-2,6-anhydro-5,7-*O*-benzylidene-l-deoxy-d-glycero-l-ido-heptitol **17**:

Compound **16** (290 mg, 0.539 mmol) were dissolved in dry MeOH (10 mL) to which NaOMe (0.5 M in MeOH, 50 mL) was added and the solution was allowed to stir at room temperature for 2 h. The solution was neutralized with DOWEX 50W H^+^, filtered, and evaporated in vacuo to furnish pure compound **17** (240 mg, 98%) as amorphous white powder. [α]_D_^25^ + 11.9 (*c* 1.4 in CHCl_3_), ^1^H NMR (400 MHz, CDCl_3_) δ: 8.32 (d, 1H, *J* 8.4 Hz, ArH), 8.11 (d, 1H, *J* 8.0 Hz, ArH), 7.55–7.46 (m, 3H, ArH), 7.36–7.29 (m, 3H, ArH), 7.21 (t, 2H, *J* 7.6 Hz, ArH), 6.97–6.88 (m, 1H, NH), 6.82 (t, 1H, *J* 8.0 Hz, ArH), 5.51 (s, 1H, CHPh), 4.30–4.21 (m, 3H, CH_2_NH, H-6a, H-2), 4.07–3.99 (m, 2H, H-1, H-6b), 3.93–3.86 (m, 2H, H-3, H-4), 3.73 (s, 1H, H-5), 3.43–3.37 (m, 1H, CH_2_NH). ^13^C NMR (100 MHz, CDCl_3_) δ: 171.5, 161.6, 159.0, 138.0, 131.9, 129.1, 128.6, 128.3, 127.0, 126.3, 126.2, 126.1, 125.3, 120.94, 120.88, 108.6, 108.4, 100.7, 76.3, 74.1, 69.8, 69.2, 66.7, 65.0, 40.9. HRMS calcd for C_25_H_24_FNO_6_Na (M + Na)^+^: 476.1485, found: 476.1489.

l-(4-Fluoro-naphth-1-amido)-3-*O*-acetyl-2,6-anhydro-4-azido-5,7-*O*-benzylidene-l,4-dideoxy-d-glycero-l-manno-heptitol **18**:

Compound **17** (250 mg, 0.551 mmol) was dissolved in in dry CH_2_Cl_2_ (10 mL) followed by addition of pyridine (89 µL, 1.10 mmol) and AcCl (39 µL, 0.551 mmol) to a stirred solution at room temperature under N_2_ atmosphere. After 2 h, the mixture was washed with NaHCO_3_ (2 × 10 mL) and brine (10 mL). The combined aqueous phases were extracted with CH_2_Cl_2_ (2 × 10 mL). The organic phases were collected and dried over Na_2_SO_4_ and evaporated. The reaction mixture was directly dissolved in dry CH_2_Cl_2_ (10 mL) and cooled to −20 °C followed by dropwise addition of Tf_2_O (182 µL, 1.10 mmol). The reaction was stirred for 2 hr and then allowed to reach room temperature. The mixture was then washed with 5% HCl (2 × 10 mL) and brine (10 mL). The combined aqueous phases were extracted with CH_2_Cl_2_ (2 × 10 mL). The collected organic phases were dried over Na_2_SO_4_ and concentrated to give crude product. The crude product (157 mg, 0.250 mmol) was dissolved in DMSO (10 mL), followed by the addition of NaN_3_ (48 mg, 0.75 mmol). The solution was stirred at 60 °C for 8 h. Then the solution was diluted with water and washed with ethyl acetate (2 × 10 mL). The collected organic layer was dried over Na_2_SO_4_ followed by filtration and evaporation in vacuo to furnish crude compound **18**, which was then purified by column chromatography using (SiO_2_, hexane: EtOAc 1:1) to afford pure compound **18** (19 mg, 15%). [α]_D_^25^ + 4.9 (*c* 0.44 in CHCl_3_), ^1^H NMR (400 MHz, CDCl_3_) δ: 8.32–8.36 (m, 1H, ArH), 8.12–8.10 (m, 1H, ArH), 7.56–7.52 (m, 3H, ArH), 7.44–7.41 (m, 2H, ArH), 7.36–7.26 (m, 3H, ArH), 6.95 (t, 1H, *J* 8.0 Hz, ArH), 6.52 (t, 1H, *J* 5.6 Hz, NH), 5.60 (s, 1H, CHPh), 5.48 (t, 1H, *J* 10.0 Hz, H-2), 4.39 (d, 1H, *J* 2.8 Hz, H-4), 4.31 (dd, 1H, *J* 12.5, 1.5 Hz, H-6a), 4.08 (dd, 1H, *J* 12.5, 1.6 Hz, H-6b), 3.97–3.92 (m, 1H, CH_2_NH), 3.75–3.62 (m, 2H, H-1, CH_2_NH), 3.52 (d, 1H, *J* 1.0 Hz H-5), 3.41 (dd, 1H, *J* 10.3, 2.3 Hz, H-3), 2.21 (s, 3H, CH_3_). ^13^C NMR (100 MHz, CDCl_3_) δ: 170.1, 169.0, 137.1, 132.0, 129.2, 128.4, 128.3, 126.9, 126.1, 125.8, 125.70, 125.67, 125.65, 120.82, 120.77, 108.7, 108.5, 101.2, 76.1, 70.1, 69.4, 66.8, 62.5, 41.1, 40.0. HRMS calcd for C_27_H_25_FN_4_O_6_Na (M + Na)^+^: 543.1656, found: 543.1658.

l-(4-Fluoro-naphth-1-amido)-2,6-anhydro-4-[4-(3,4,5-trifluorophenyl)-1H-1,2,3-triazol-1-yl]-l,4-dideoxy-d-glycero-l-manno-heptitol **19**:

A solution of compound **18** (19 mg, 0.036 mmol) in 90% aq AcOH (10 mL) was stirred at 90 ^0^C for 3 h. The solvents were evaporated and co-evaporated with toluene to remove residual AcOH. The residue was dissolved in MeOH (10 mL), to which NaOMe in MeOH (0.5 M, 1 mL) was added and the solution was stirred at room temperature for 5 h. The solution was then neutralized by DOWEX 50 W H^+^ resin, filtered, and evaporated. The residue (18 mg, 0.046 mmol) was dissolved in DMF (10 mL), followed by the successive addition of 3,4,5-trifluorophenyl acetylene (21 mg, 0.138 mmol) and CuI (8 mg, 0.1 mmol). After 5 min, Et_3_N (6.3 μL, 0.046 mmol) was added and the mixture was stirred at room temperature for 48 h. The solvent was evaporated in vacuo to furnish crude 18, which was purified by column chromatography (SiO_2_, hexane: EtOAc 1:12) to afford pure **19** (10.6 mg, 42%). [α]_D_^25^ + 2.5 (*c* 1.1 in MeOH), ^1^H NMR (400 MHz, CD_3_OD) δ: 8.51 (s, 1H, triazole H), 8.33–8.31 (m, 1H, ArH), 8.16–8.14 (m, 1H, ArH), 7.71–7.62 (m, 5H, ArH), 7.28 (t, 1H, *J* 8.0 Hz, ArH), 4.86 (d, 1H, *J* 3.2 Hz, H-1), 4.30 (dd, 1H, *J* 10.4, 9.0 Hz, H-2), 4.18 (dd, 1H, *J* 11.7, 1.7 Hz, CH_2_NH), 4.12 (d, 1H, *J* 3.2 Hz, H-4), 3.84–3.77 (m, 2H, H-5, H-6a), 3.72–3.62 (m, 3H, H-6b, CH_2_NH, H-1). ^13^C NMR (100 MHz, CD_3_OD) δ: 172.1, 133.30, 133.25, 132.13, 132.09, 129.1, 128.0, 127.1, 126.7, 125.0, 124.9, 122.9, 121.5, 121.4, 110.9, 110.7, 109.7, 109.5, 81.2, 80.7, 70.1, 69.2, 67.2, 62.9, 42.5. HRMS calcd for C_26_H_23_F_4_N_4_O_5_ (M + H)^+^: 547.1605, found: 547.1614.

Fluorescence polarization experiments:

Fluorescence polarization experiments were carried out either with a POLARStar plate reader and FLUOstar Galaxy software or with a PheraStarFS plate reader and PHERAstar Mars version 2.10 R3 software (BMG, Offenburg, Germany). The dissociation constant (Kd) values were determined in PBS as described earlier [12,13]. Specific conditions for galectin-1, 3, 4N, 4C, 7, 8N, 9N, and 9C were kept as reported [32,33]. Average Kd values and SEMs were calculated from 2–8 single-triple point measurements showing between 30% and 70% inhibition.

Co-crystallization of galectin-3 C-terminal domain with compounds **14** and **19:**

Galectin-3C [34] (C-terminal domain) solution (12 µL, 20 mg/mL in 10 mM sodium phosphate buffer pH 7.5, 100 mM NaCl and 10 mM β-mercaptoethanol) was mixed with either compound **14** or **19** (1.2 µL, 50 mM in 100% DMSO) and incubated on ice for 30 min. Crystallization drops of 2 + 2 µL were set up over 1 mL reservoir solution (30% PEG 4000, 0.1 M Tris/HCl pH 7.5, 0.1 M MgCl_2_, 0.4 M NaSCN, 7.9 mM β-mercaptoethanol). Immediately after setup, the drops were seeded by adding 0.25 µL of crystals of apo-galectin-3C that had been crushed using Seed Beads (Hampton Research) in a stabilizing solution (reservoir solution). Co-crystals of compound **14** measuring 0.03 × 0.05 × 0.05 mm and compound **19** measuring 0.1 × 0.15 × 0.15 mm were flash-frozen in cryo solution (15% glycerol, 25.5 *w*/*v* % PEG 4000, 250 mM NaSCN, 85 mM Tris/HCl pH 7.5, 85 mM MgCl_2_, 2.5 mM **14** or 0.5 mM **19**).

Data collection and structure solution of galectin-3C in complex with **13** and **18:**

Data to 1.5 Å for compound **14** were collected at 100 K at station I911-3 of MAX-lab, Lund, Sweden (λ = 1.0000 Å), equipped with a marMosaic 225mm CCD detector. Data to 1.4 Å for compound **19** were collected at station I911-2 of the same synchrotron (λ = 1.0384 Å), equipped with a marCCD 165 mm detector. Data for both structures were integrated using XDS and scaled using XSCALE [35]. The structures were refined using Refmac5 [36] with PDB entry 3ZSL [31] as starting model, first by rigid-body refinement. 5% of the total reflections were randomly set aside for cross validation. The graphic software Coot [37] has been used for model building and Monomer Library Sketcher software [38] for generation of restraints for the ligands **14** and **19**. The models were refined to a resolution of 1.5 Å/1.4 Å with anisotropic B-factors. Water molecules were added to positive difference density peaks more than 5 σ above the mean and present in the 2m|Fo|-d|Fc| map at 1 σ level. Refinement statistics are listed in Table S1. Molecular images were generated using PyMOL v1.7 (Schrödinger LLC, New York, NY, USA).

## Figures and Tables

**Table 1:** Dissociation constants (K_d_, µM) of methyl β-d-galactopyranosyls [14], aldoxime **1**, oxazole **2**, alkene **3**, and amides **4,** and **7**–**11** against galectin-3 determined in a competitive fluorescence anisotropy assay [12,13].

Me β-gal	1	2	3	4	7	8	9	10	11
4400	370 [7,8]	90 [9]	1600 ± 90 ^1^	39 ± 2.3	22 ± 0.75	79 ± 7.8	43 ± 0.38	28 ± 0.81	22 ± 0.79

^1^ Dissociation constant averages and SEM were calculated from two to eight single-point measurements showing between 20% and 80% inhibition.

**Table 2:** Dissociation constants (K_d_, µM) of compounds **14** and **19** against galectin-1, 3, 4N, 4C, 7, 8N, 9N, and 9C determined in a competitive fluorescence anisotropy assay.

	Galectin							
	1	3	4N ^1^	4C ^2^	7	8N ^1^	9N ^1^	9C ^2^
**14**	220 ± 19 ^3^	2.0 ± 0.18	870 ± 170	100 ± 43	ND ^4^	1300 ± 150	>2000	ND
**19**	23 ± 1.4	0.54 ± 0.060	31 ± 0.80	1.3 ± 0.039	>500	1000 ± 270	14 ± 0.89	130 ± 6
Me β-gal	>10,000 [14]	4400 [14]	6600 [23]	10,000 [23]	4800 [14]	6300 [14]	3300 [14]	8600 [24]

^1^*N*-terminal domain ^2^ C-terminal domain. ^3^ Dissociation constant averages and SEM were calculated from two to eight single-point measurements showing between 20% and 80% inhibition. ^4^ Not determined.

**Table 3:** Data processing and refinement statistics for the X-ray crystal structures. Values in parentheses are for the highest resolution shell, unless noted otherwise. rmsd = root mean square deviation; $R_{merge}=\;\frac{\sum_{hkl}\sum_{i}\left|\bar{|F_{hkl}}\right|-|F_{hkl,i}||}{\sum_{hkl}\sum_{i}|F_{hkl,i}|}$, where $\left|\bar{F_{hkl}}\right|$ is the mean of all observations $\left|F_{hkl,i}\right|$ of a given reflection *hkl* and the sum is over all reflections and observations; $R_{model}=\frac{\sum_{hkl}|\left|F_{o,hkl}\right|-\left|F_{c,hkl}\right||}{\sum_{hkl}\left|F_{o,hkl}\right|}$, where $\left|F_{o,hkl}\right|$ and $\left|F_{c,hkl}\right|$ are respectively the observed structure factors and the ones calculated from the model for a given reflection *hkl* and the sum is over all reflections *hkl*. The bias-free R_free_ was calculated on 5% of the data that were excluded from the refinement calculations.

Compound	14	19
PDB code	6TF6	6TF7
station	I911-3	I911-2
wavelength (Å)	1.000	1.03841
unit cell (Å)	a = 35.85 b = 57.34 c = 62.04	a = 36.14 b = 58.16 c = 62.84
Space group	P2_1_2_1_2_1_	P2_1_2_1_2_1_
resolution range (Å)	31.0–1.5 (1.54–1.50)	29.1–1.4 (1.44–1.4)
completeness (%)	98.8 (96.3)	99.6 (96.8)
Total reflections unique reflections	74,804 (6175) 20,755 (1522)	155,192 (7866) 26,601 (1923)
multiplicity	3.6 (3.4)	5.8 (4.2)
R_merge_ (%)	0.073 (0.725)	0.048 (0.519)
mean I/σ(I)	12.6 (1.8)	24.0 (2.8)
Wilson B-factor (Å^2^)	15.6	14.6
refinement program	Refmac5	Refmac5
R_model_ (F; %)	0.116 (0.173)	0.135 (0.240)
R_free_ (F; %)	0.174 (0.275)	0.187 (0.324)
reflections used in refinement (for R_free_)	19,717 (1374) 1038 (72)	25,272 (1767) 1330 (93)
average B-factors (Å^2^)	protein: 14.2 ligand: 21.5 solvent: 33.4	protein: 13.5 ligand: 19.4 solvent: 28.9
Ramachandran outliers (%)	1	0
rotamer outliers (no. and %) MolProbity clash score	1, 0.6% 2.48	0, 0.0% 2.12
bond length rmsd from ideal (Å) bond angle rmsd from ideal (°)	0.020 2.1	0.019 2.2

## Supplementary Materials

The following are available online, Copies of ^1^H and ^13^C nmr spectra, Table S1: Data processing and refinement statistics for the X-ray crystal structures of **14** and **19** in complex with galectin-3.
