# Supplementary material for: 3-Substituted 1-Naphthamidomethyl-C-galactosyls Interact with Two Unique Sub-Sites for High-Affinity and High-Selectivity Inhibition of Galectin-3
Source: Molecules. 2019 Dec 12;24(24):4554. doi: 10.3390/molecules24244554 (PMC6943516; doi:10.3390/molecules24244554)

Supporting Information

3-Substituted 1-Naphthamidomethyl-C-galactosyls Interact with Two Unique Sub-sites for High-Affinity and High-Selectivity Inhibition of Galectin-3

Alexander Dahlqvist [1,†] Santanu Mandal [1,†] Kristoffer Peterson [1] Maria Håkansson [2] Derek T. Logan [2,3] Fredrik R. Zetterberg [4] Hakon Leffler [5] and Ulf. J. Nilsson [1,*]

[1] Centre for Analysis and Synthesis, Department of Chemistry, Lund University, POB124, SE-22100, Lund, Sweden; alexander.dahlqvist@chem.lu.se (A.D.); santanuiiser@gmail.com (S.M.); kristoffer.peterson@redglead.com (K.P.)

[2] SARomics Biostructures AB, Medicon Village, SE-223 63 Lund, Sweden; maria.hakansson@saromics.com (M.H.); derek.logan@biochemistry.lu.se (D.T.L.)

[3] Biochemistry and Structural Biology, Center for Molecular Protein Science, Department of Chemistry, Lund University, POB124, SE-22100, Lund, Sweden

[4] Galecto Biotech AB, Sahlgrenska Science Park, Medicinaregatan 8 A, SE-413 46 Gothenburg, Sweden; FZ@Galecto.com

[5] Department of Laboratory Medicine, Section MIG, Lund University BMC-C1228b, Klinikgatan 28, 221 84 Lund, Sweden; hakon.leffler@med.lu.se

* Correspondence: ulf.nilsson@chem.lu.se

† These authors contributed equally and share first authorship

Table of Contents:

^{1}H NMR and ^{13}C NMR spectra of **3** S3

^{1}H NMR and ^{13}C NMR spectra of **4** S4

^{1}H NMR and ^{13}C NMR spectra of **7** S5

^{1}H NMR and ^{13}C NMR spectra of **8** S6

^{1}H NMR and ^{13}C NMR spectra of **9** S7

^{1}H NMR and ^{13}C NMR spectra of **10** S8

^{1}H NMR and ^{13}C NMR spectra of **11** S9

^{1}H NMR and ^{13}C NMR spectra of **12** S10

^{1}H NMR and ^{13}C NMR spectra of **13** S11

^{1}H NMR and ^{13}C NMR spectra of **14** S12

^{1}H NMR and ^{13}C NMR spectra of **15** S13

^{1}H NMR and ^{13}C NMR spectra of **16** S14

^{1}H NMR and ^{13}C NMR spectra of **17** S15

^{1}H NMR and ^{13}C NMR spectra of **18** S16

^{1}H NMR and ^{13}C NMR spectra of **19** S17

^{1}H NMR Spectrum of **3** in CD_3OD (400 MHz):

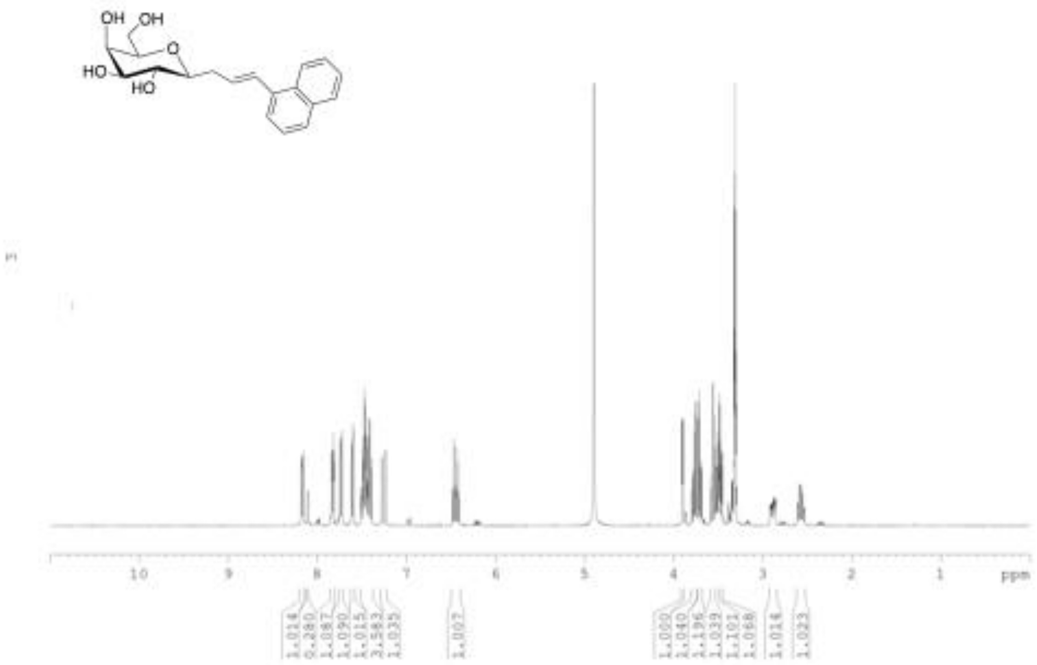

^{13}C NMR Spectrum of **3** in CD_3OD (100 MHz):

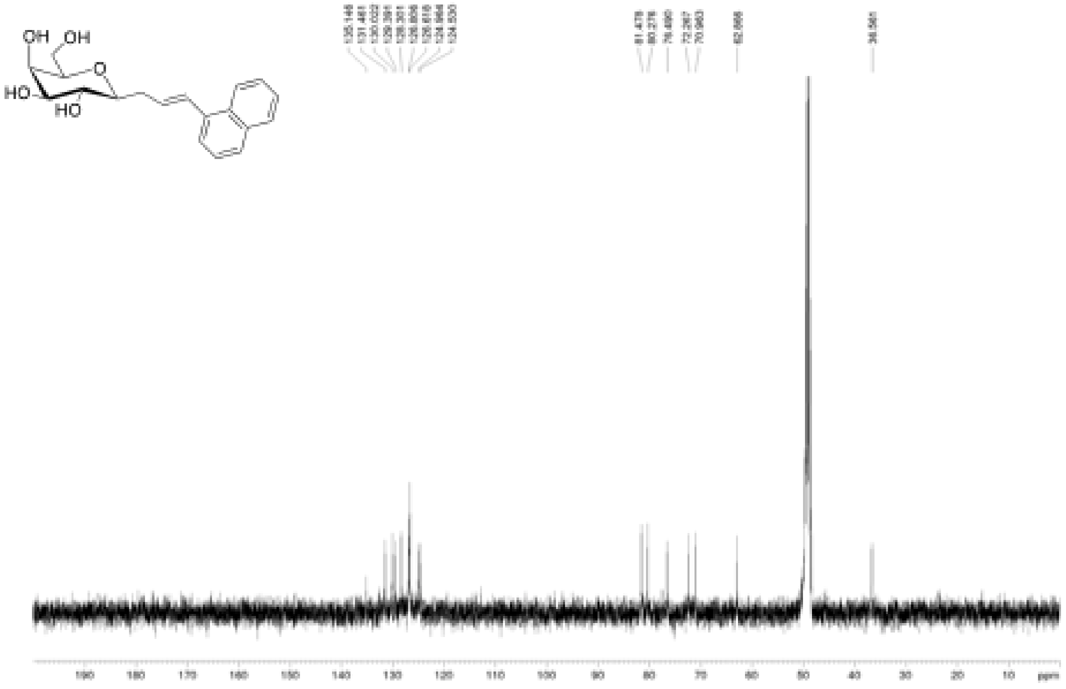

^{1}H NMR Spectrum of **4** in CD_3OD (400 MHz):

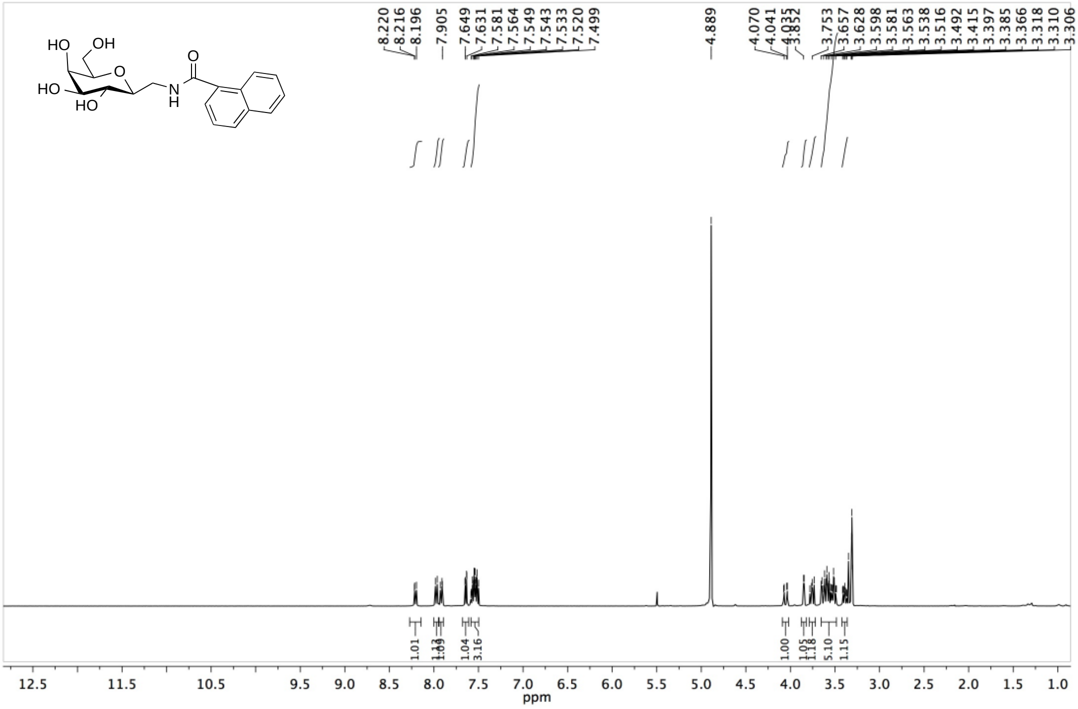

^{13}C NMR Spectrum of **4** in CD_3OD (100 MHz):

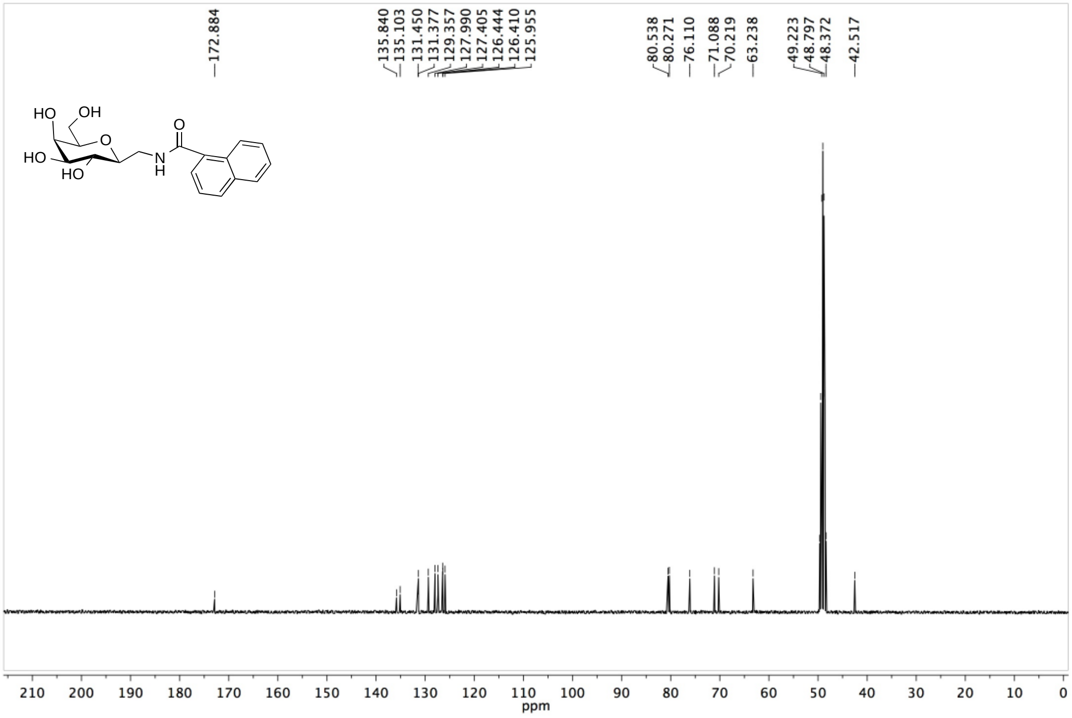

^{1}H NMR Spectrum of **7** in CD_3OD (400 MHz):

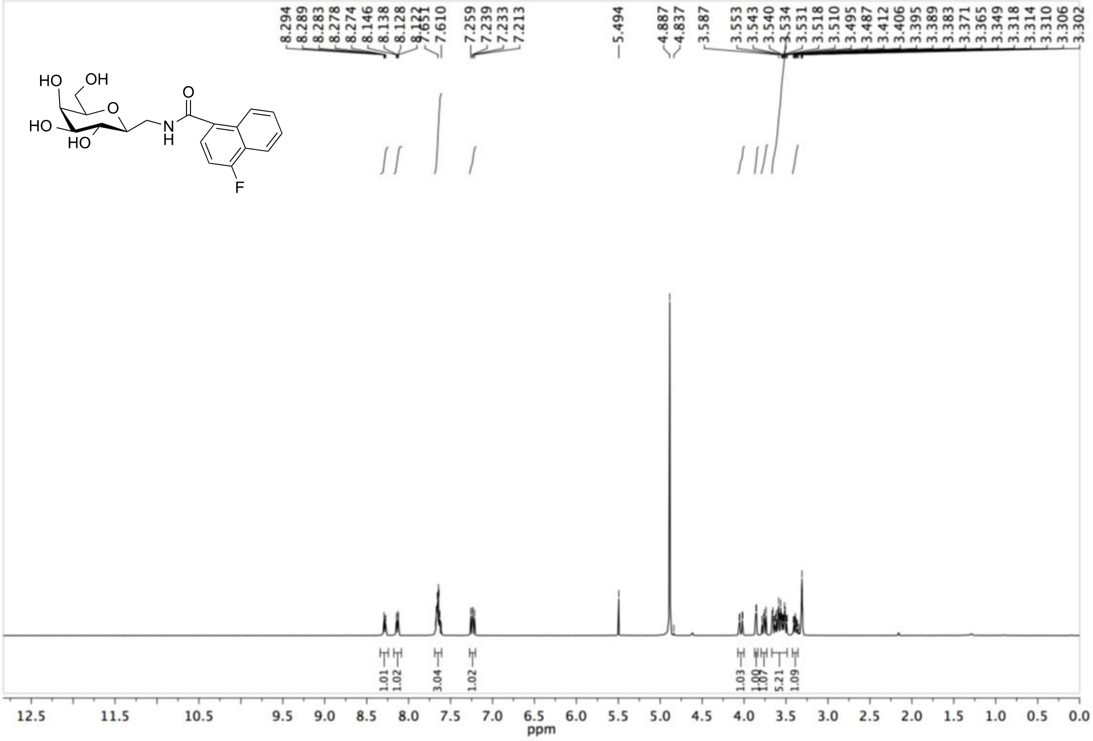

^{13}C NMR Spectrum of **7** in CD_3OD (100 MHz):

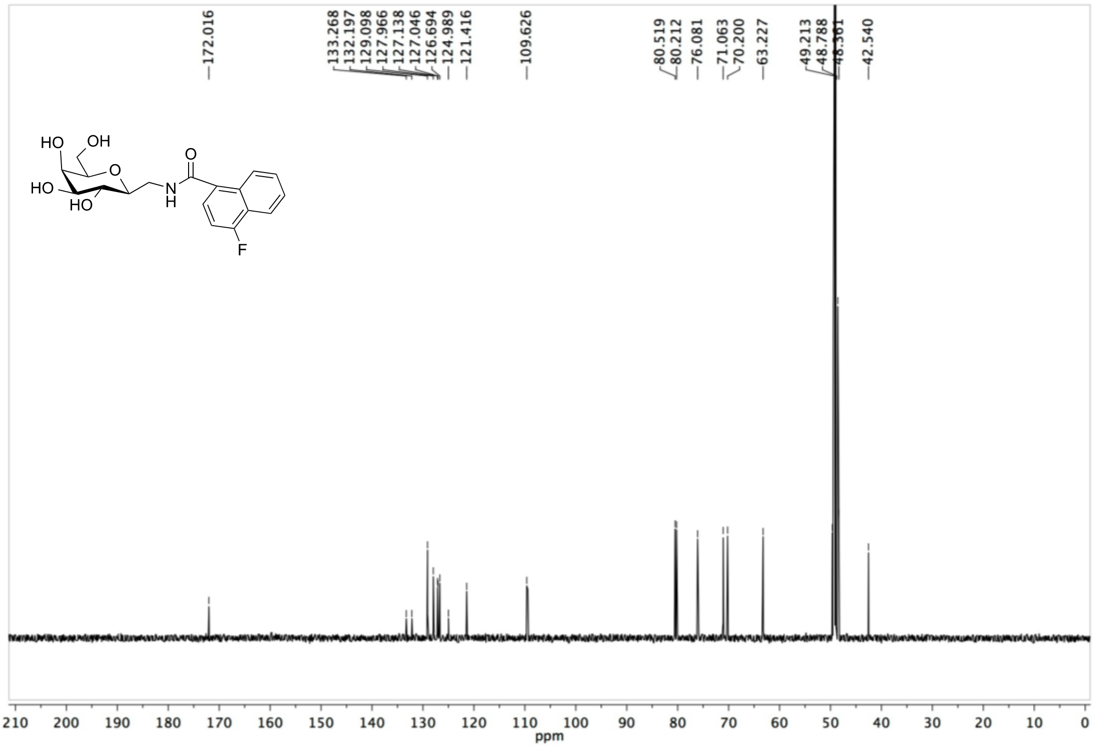

^{1}H NMR Spectrum of **8** in CD_3OD (400 MHz):

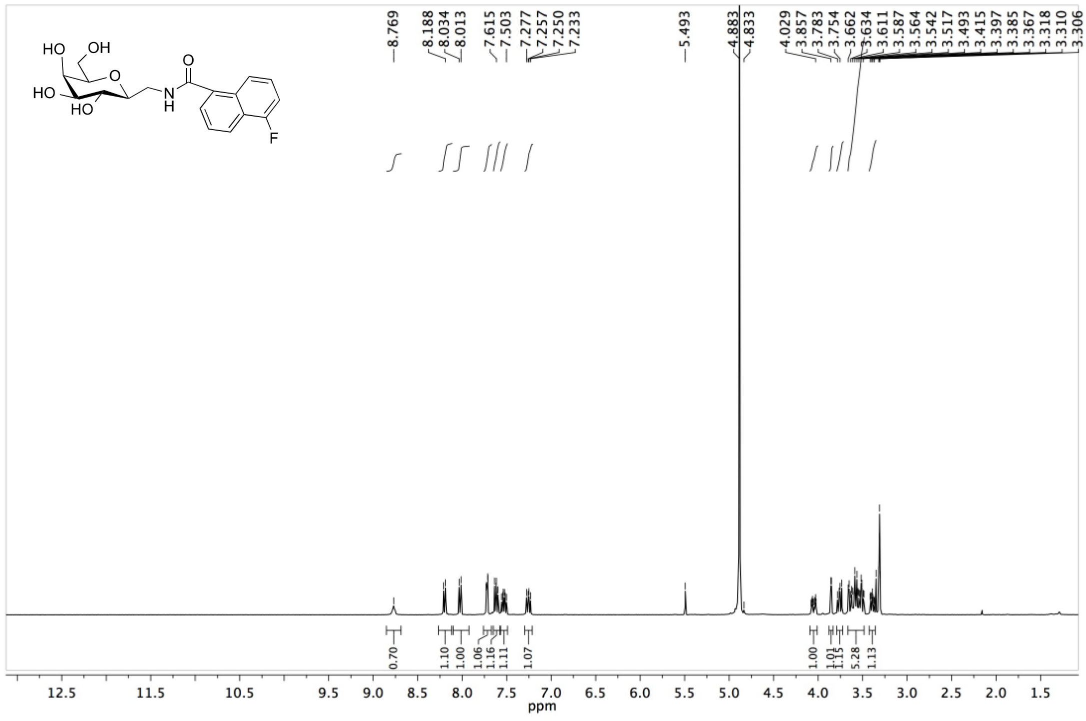

^{13}C NMR Spectrum of **8** in CD_3OD (100 MHz):

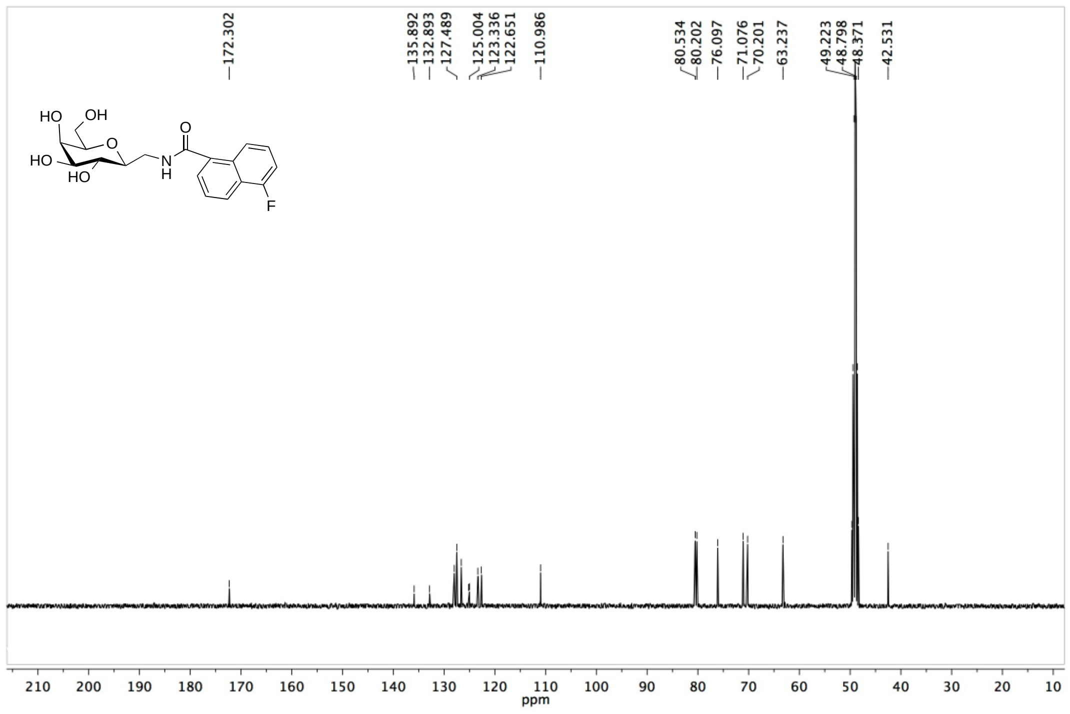

^{1}H NMR Spectrum of **9** in CD_3OD (400 MHz):

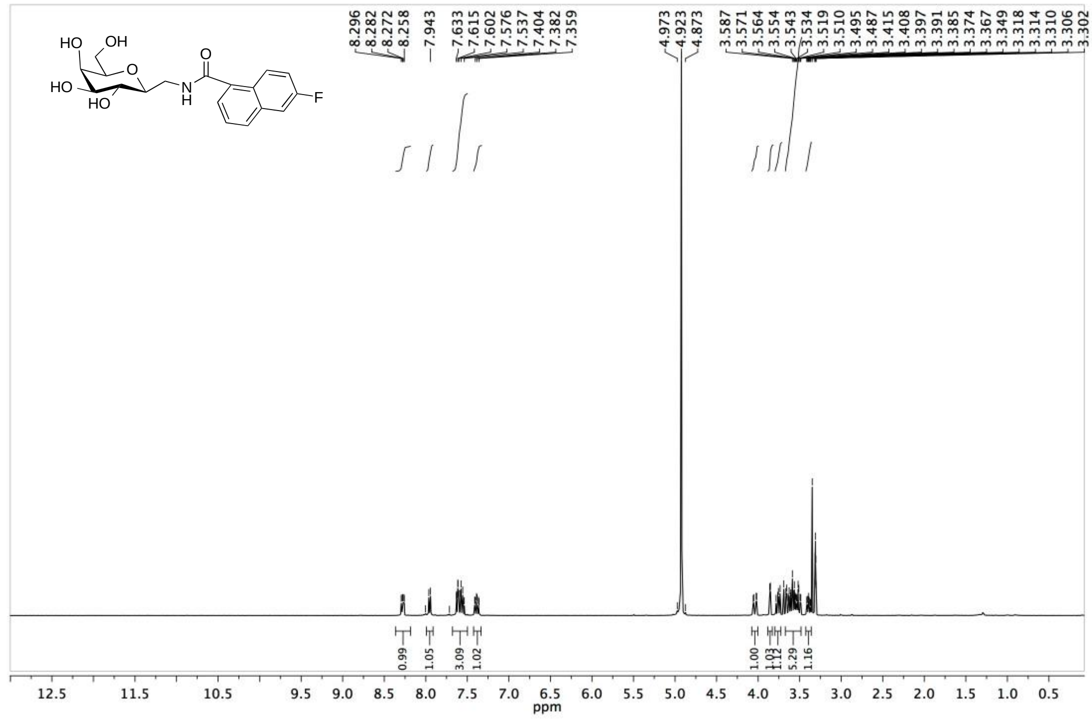

^{13}C NMR Spectrum of **9** in CD_3OD (100 MHz):

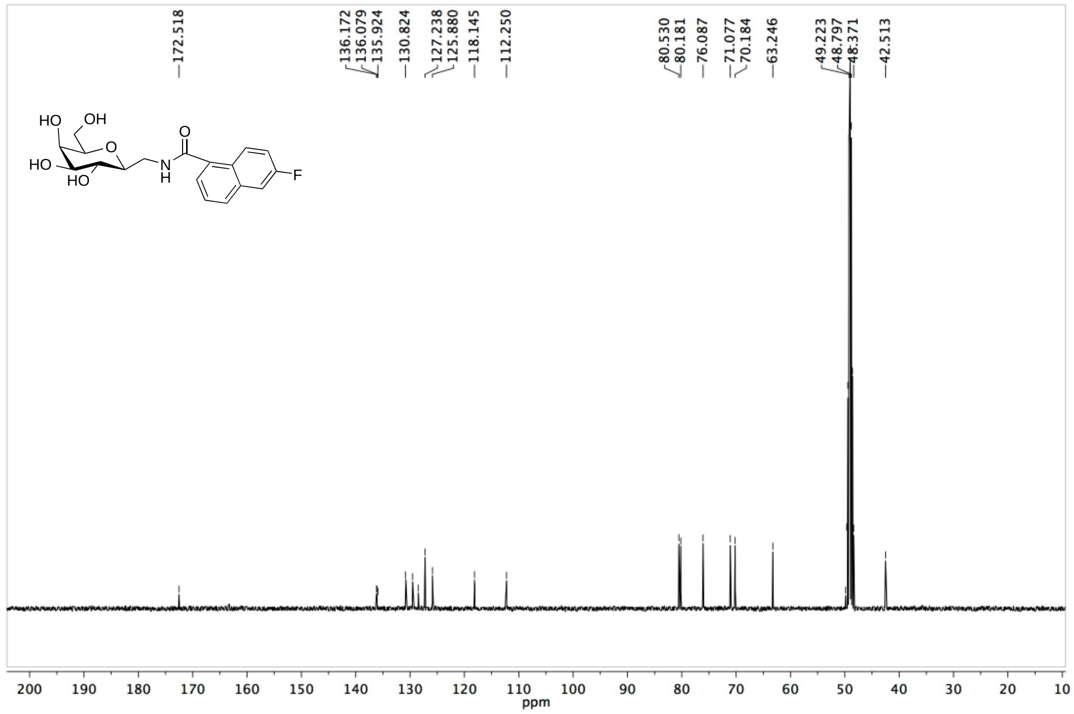

^{1}H NMR Spectrum of **10** in CD_3OD (400 MHz):

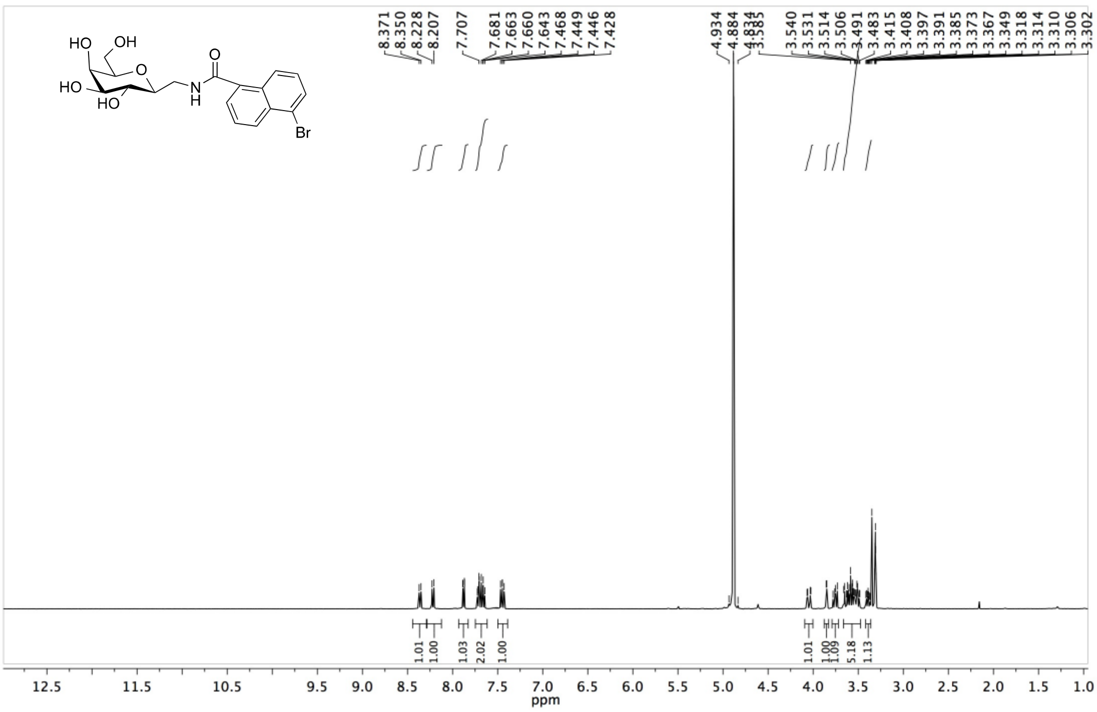

^{13}C NMR Spectrum of **10** in CD_3OD (100 MHz):

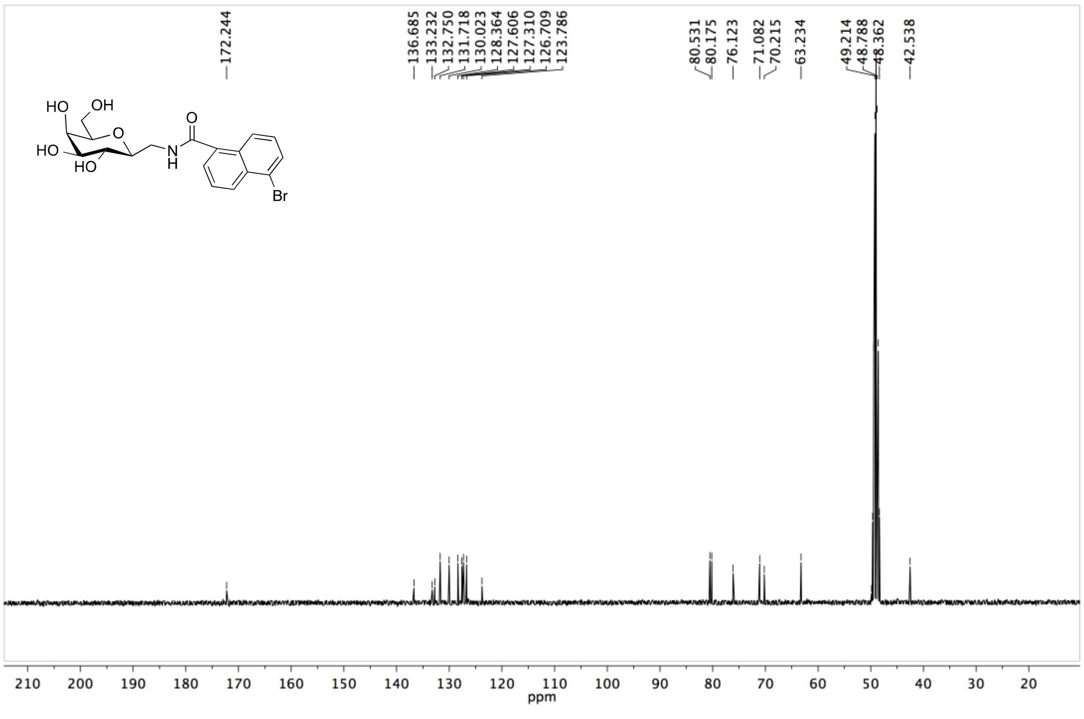

^{1}H NMR Spectrum of **11** in CD_3OD (400 MHz):

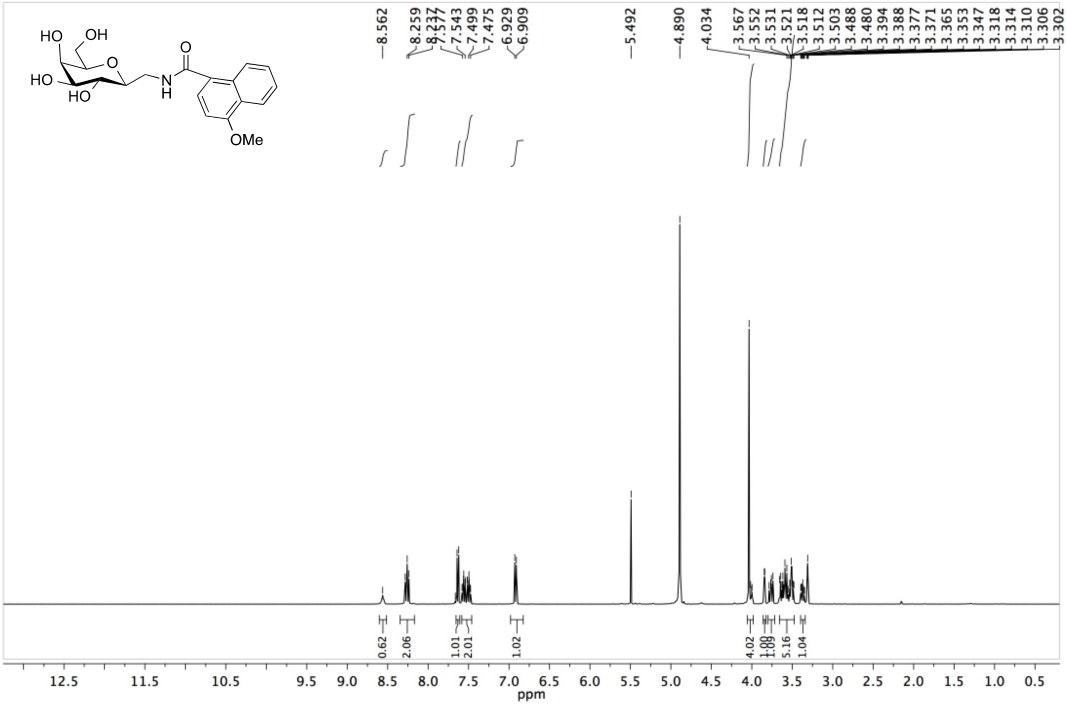

^{13}C NMR Spectrum of **11** in CD_3OD (100 MHz):

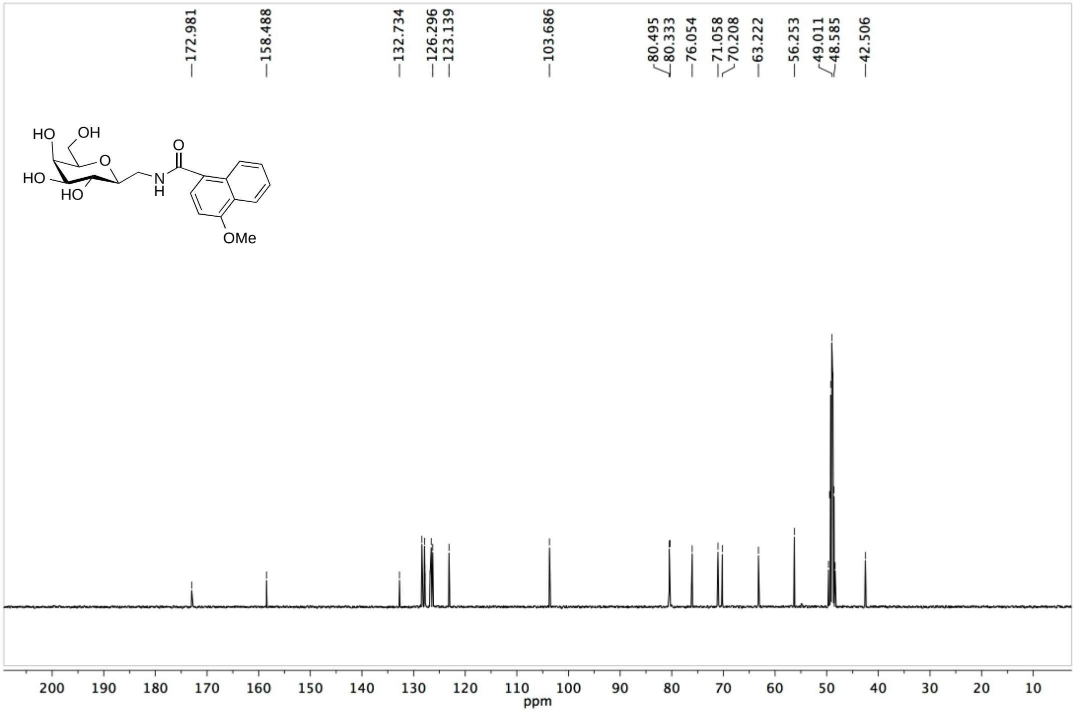

^{1}H NMR Spectrum of **12** in CD_3OD (400 MHz):

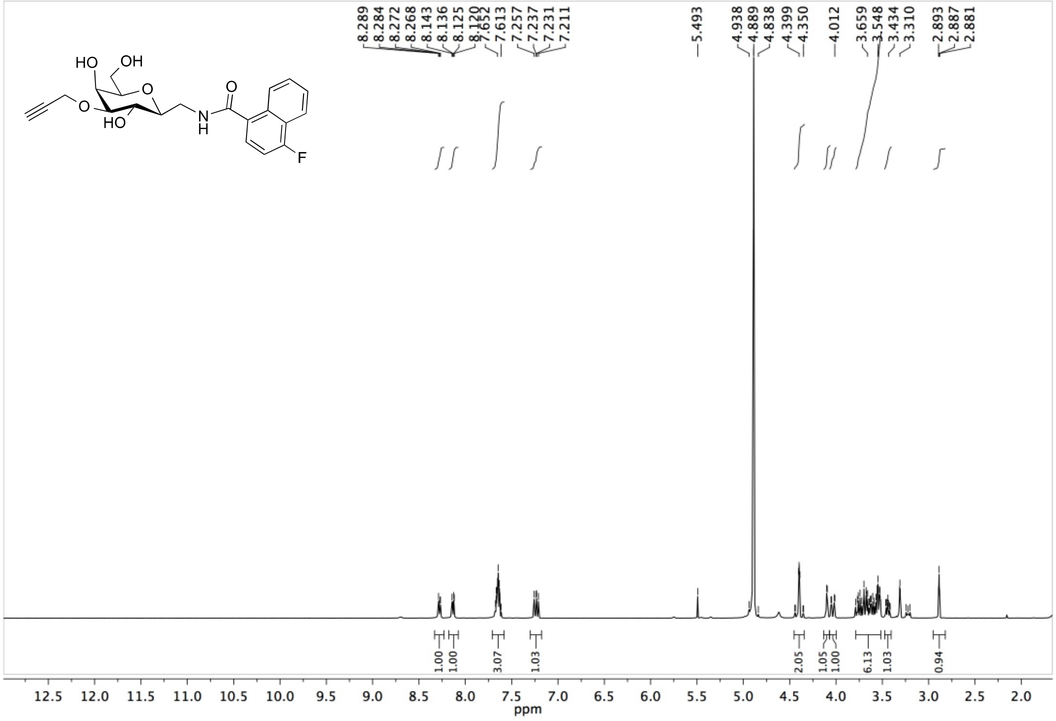

^{13}C NMR Spectrum of **12** in CD_3OD (100 MHz):

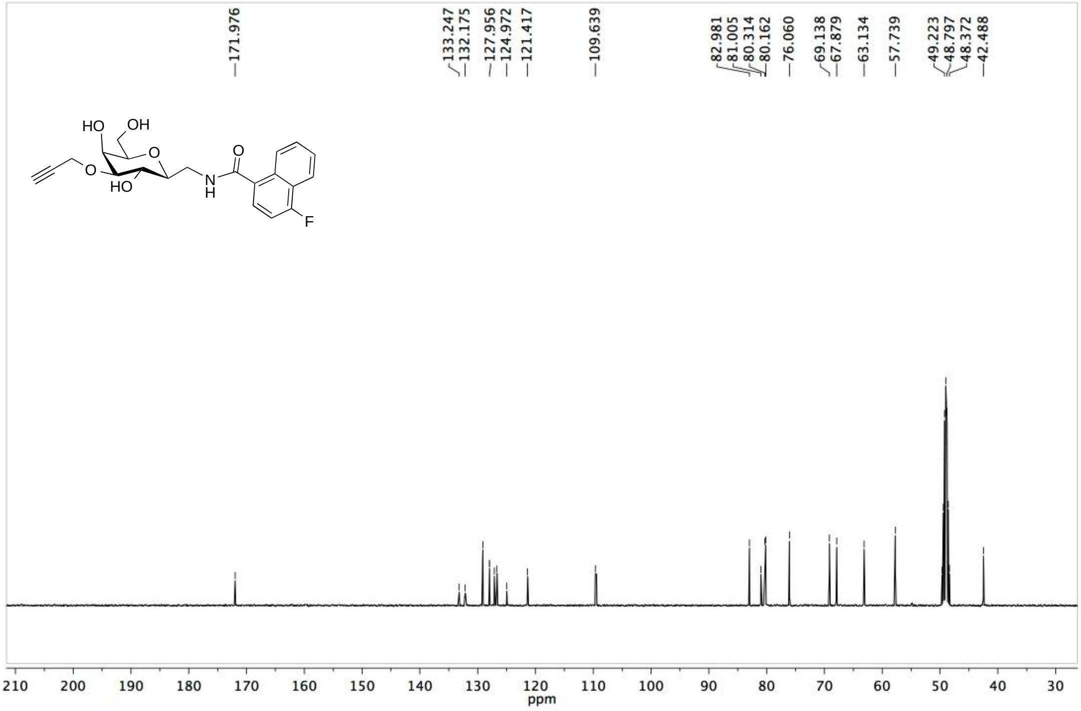

^{1}H NMR Spectrum of **13** in $CDCl_3$ (400 MHz):

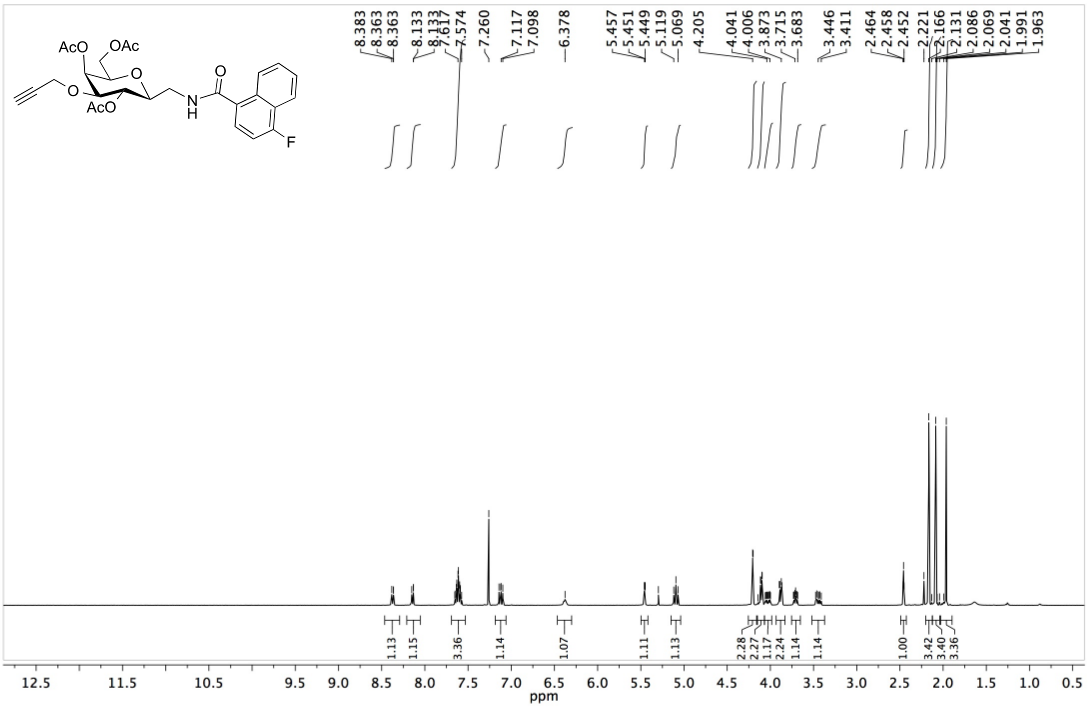

^{13}C NMR Spectrum of **13** in $CDCl_3$ (100 MHz):

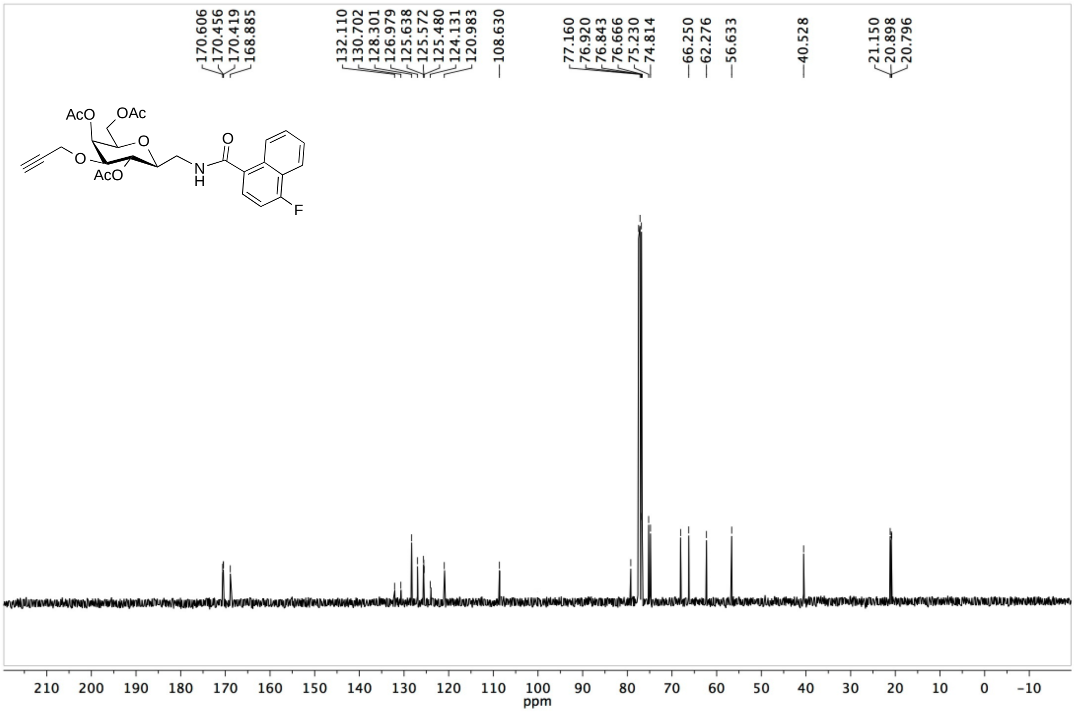

^{1}H NMR Spectrum of **14** in CD_3OD (400 MHz):

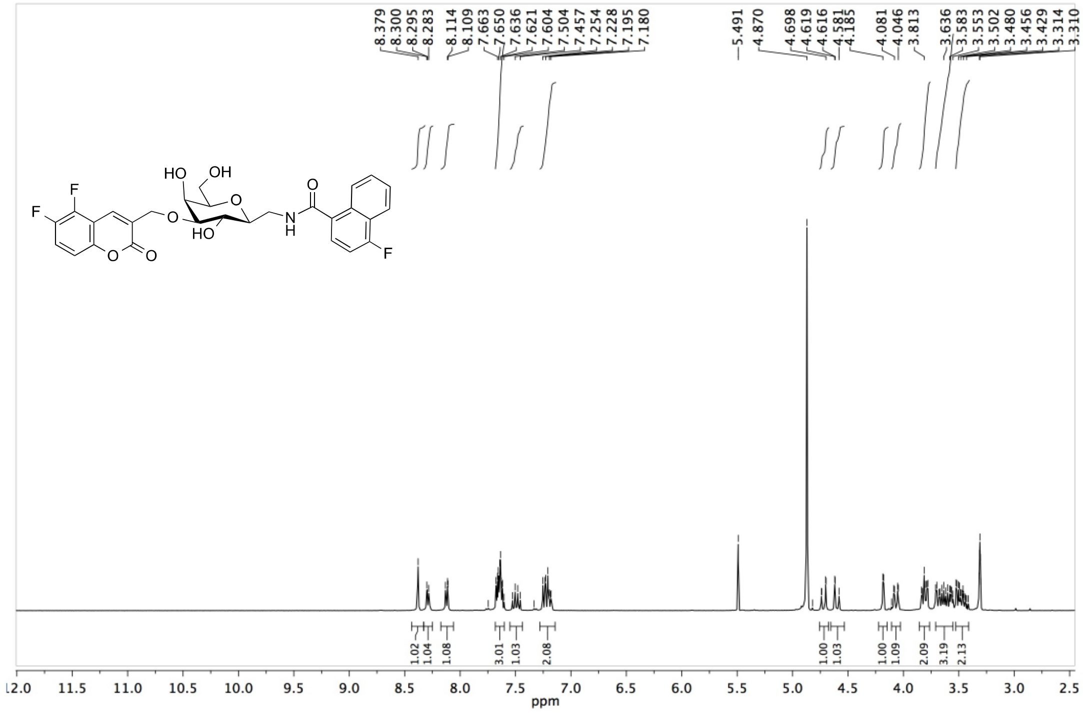

^{13}C NMR Spectrum of **14** in CD_3OD (100 MHz):

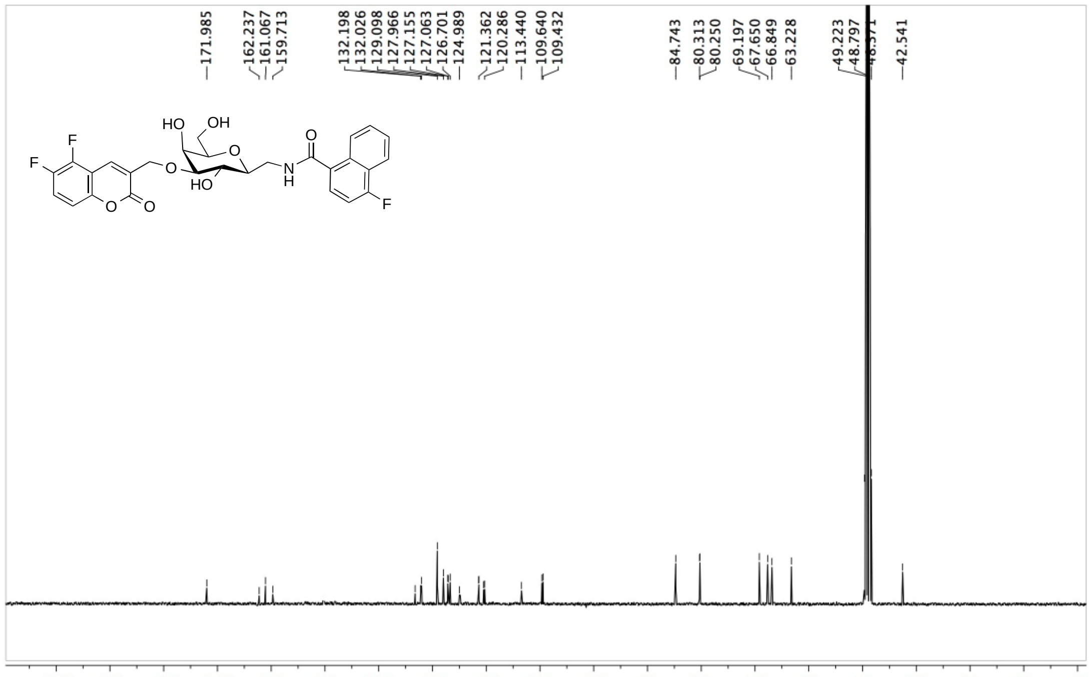

^{1}H NMR Spectrum of **15** in $CDCl_3$ (400 MHz):

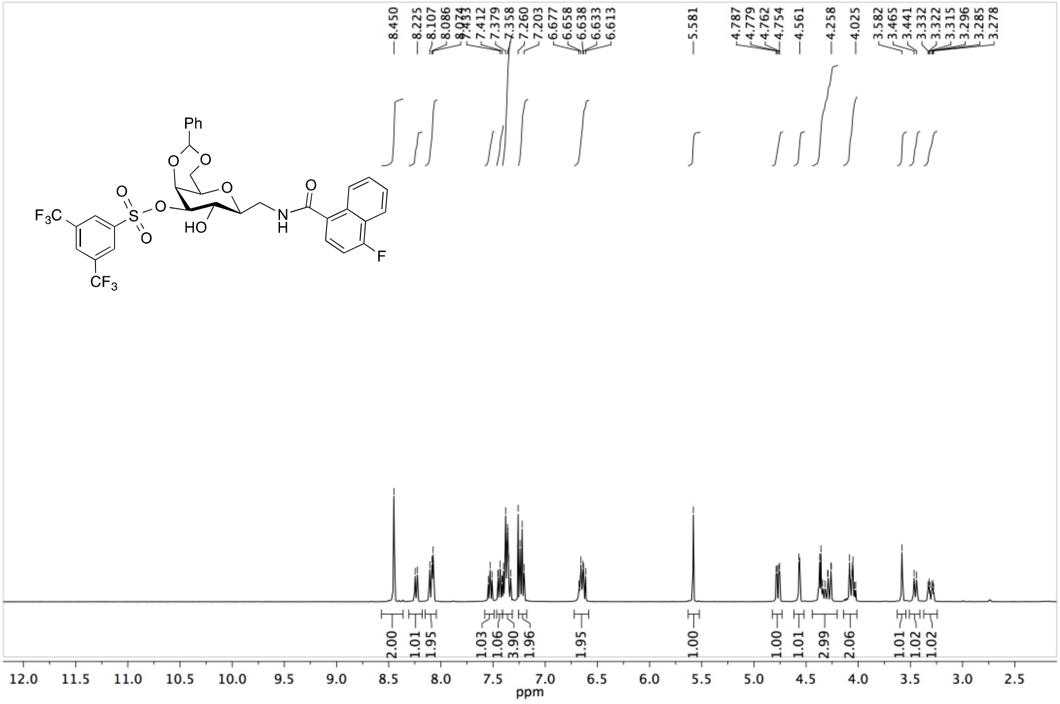

^{13}C NMR Spectrum of **15** in $CDCl_3$ (100 MHz):

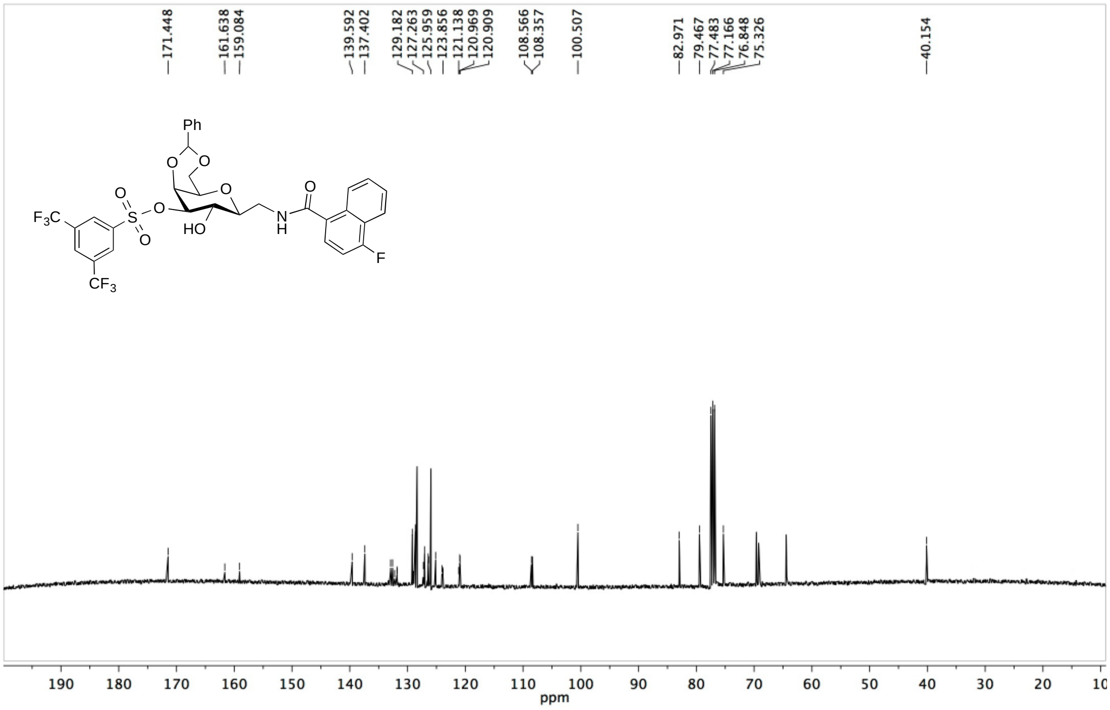

^{1}H NMR Spectrum of **16** in $CDCl_3$ (400 MHz):

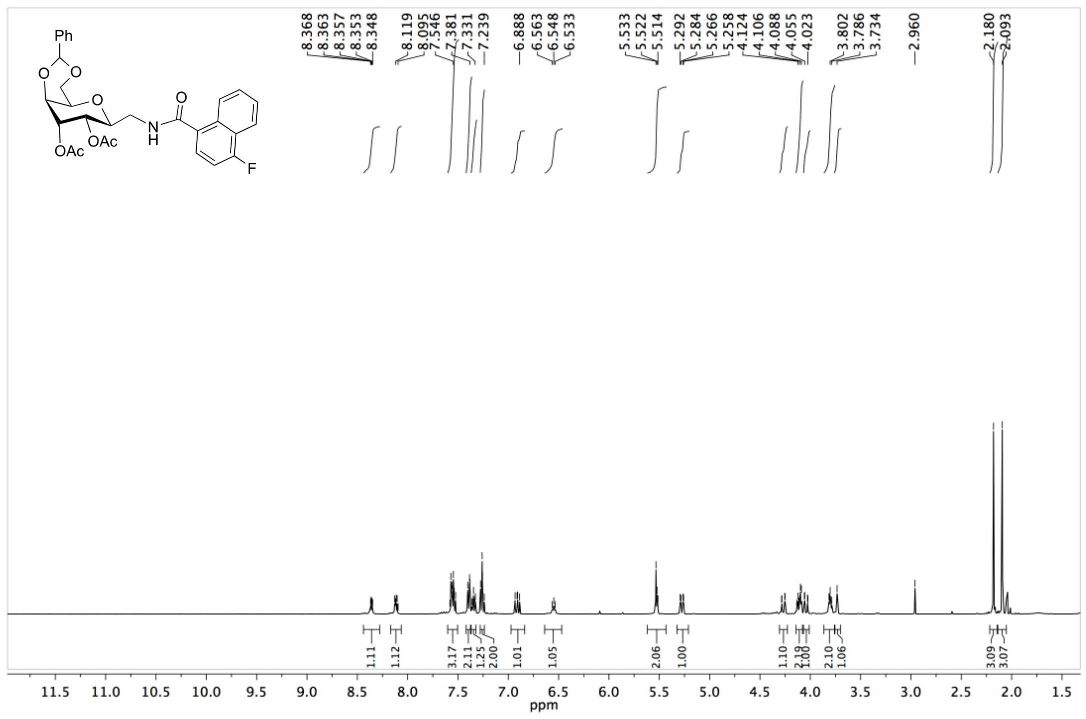

^{13}C NMR Spectrum of **16** in $CDCl_3$ (100 MHz):

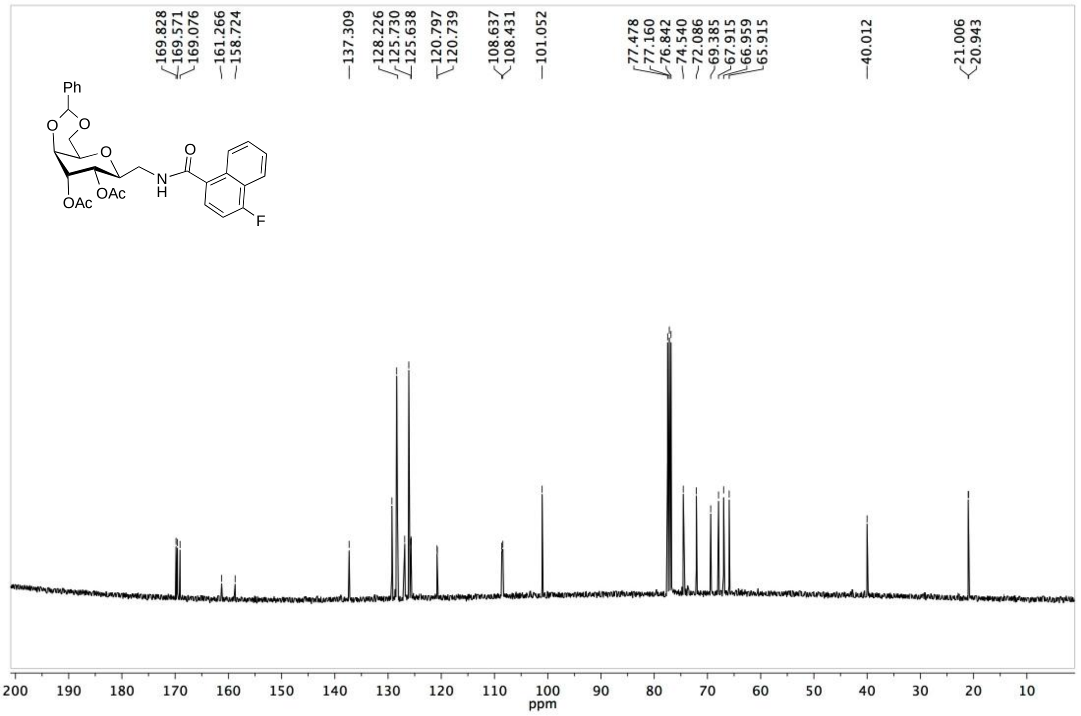

^{1}H NMR Spectrum of **17** in $CDCl_3$ (400 MHz):

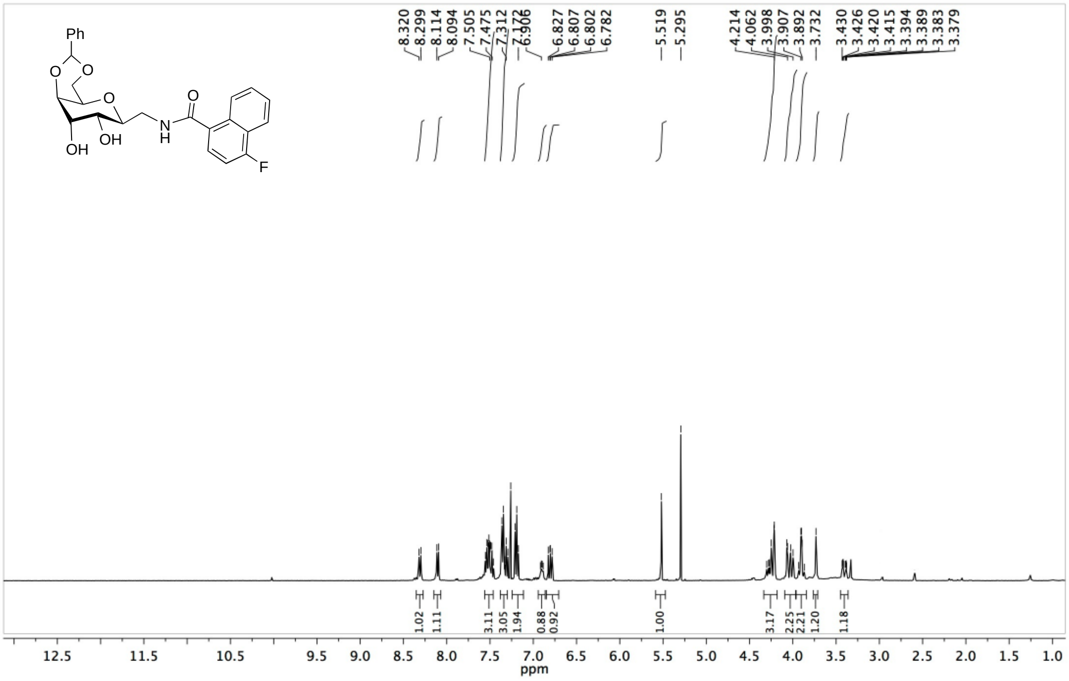

^{13}C NMR Spectrum of **17** in $CDCl_3$ (100 MHz):

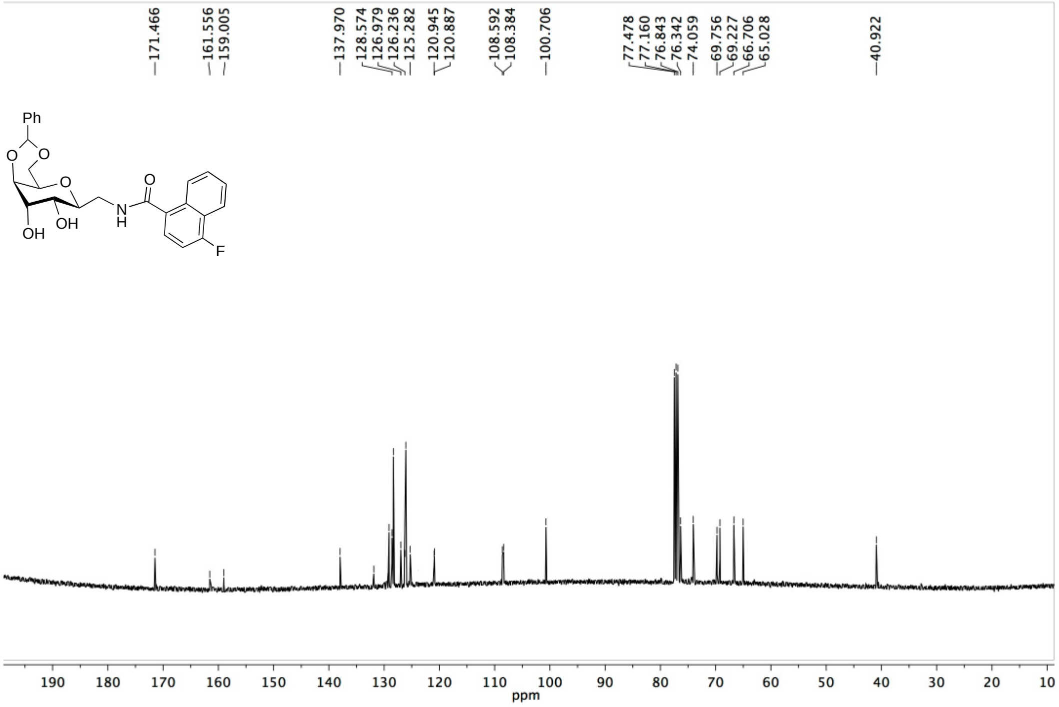

^{1}H NMR Spectrum of **18** in $CDCl_3$ (400 MHz):

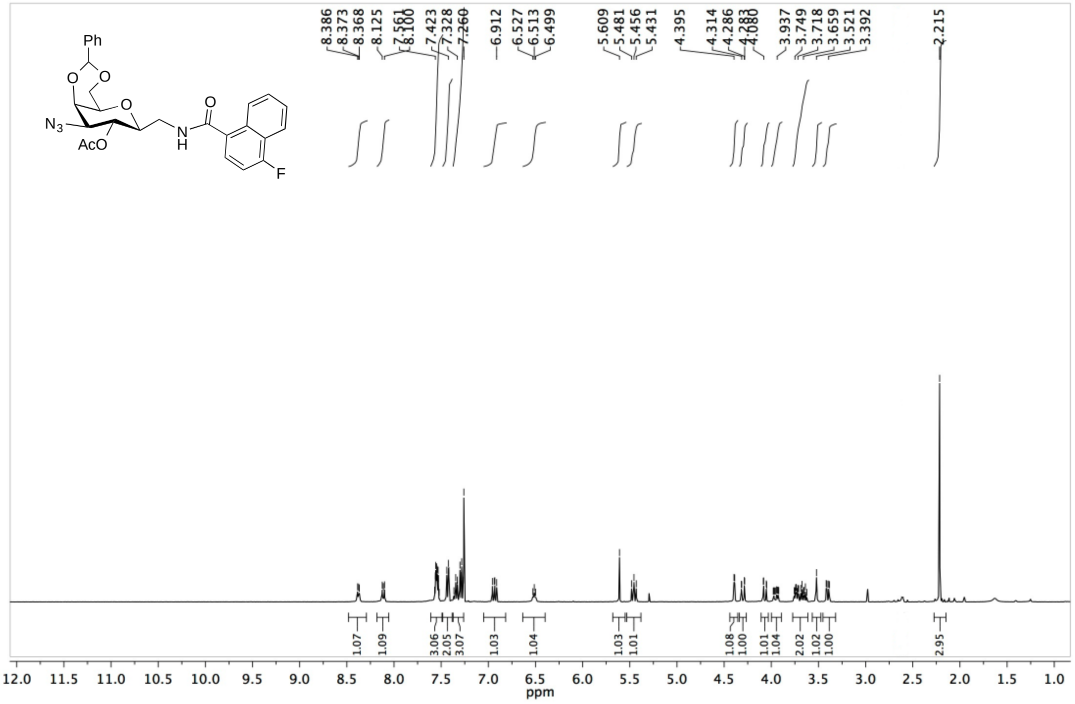

^{13}C NMR Spectrum of **18** in $CDCl_3$ (100 MHz):

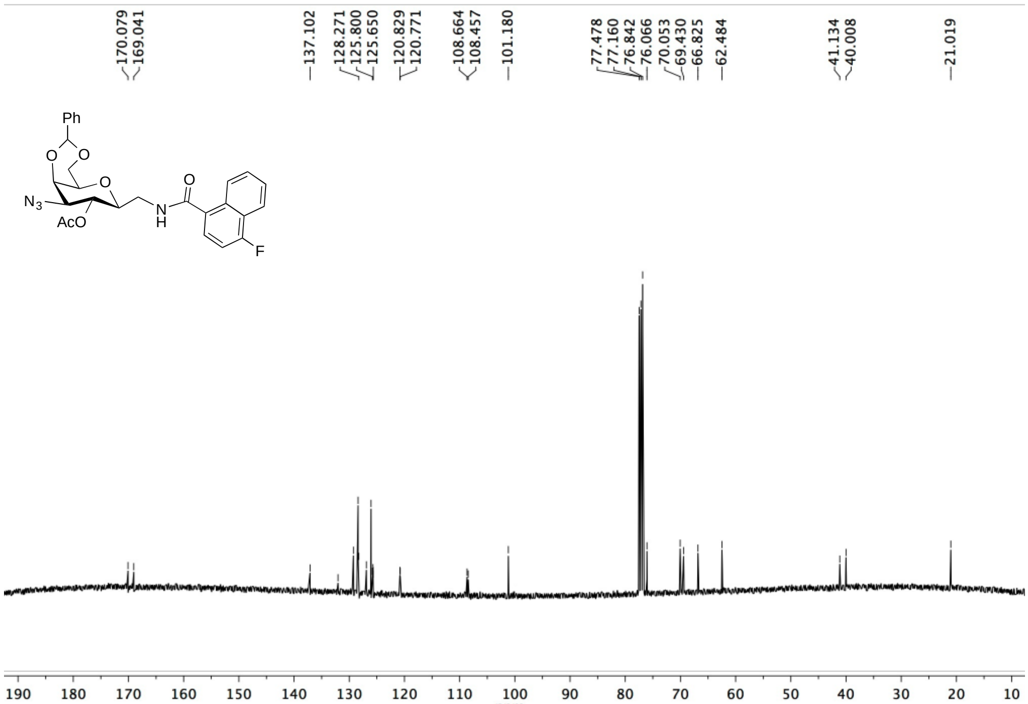

^{1}H NMR Spectrum of **19** in CD_3OD (400 MHz):

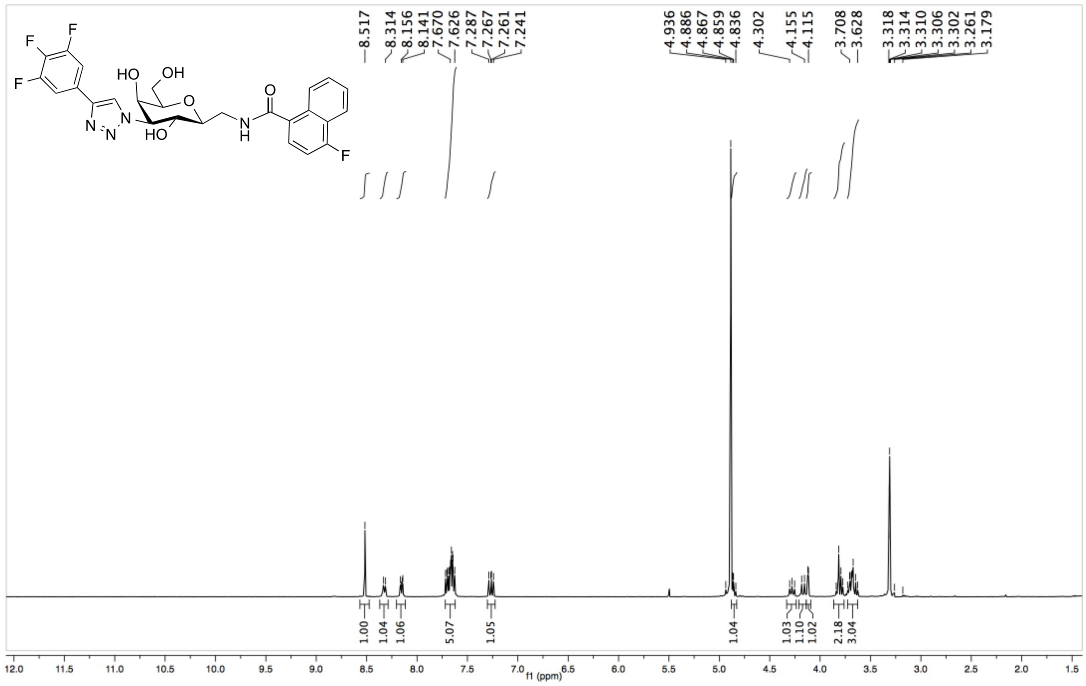

^{13}C NMR Spectrum of **19** in CD_3OD (100 MHz):

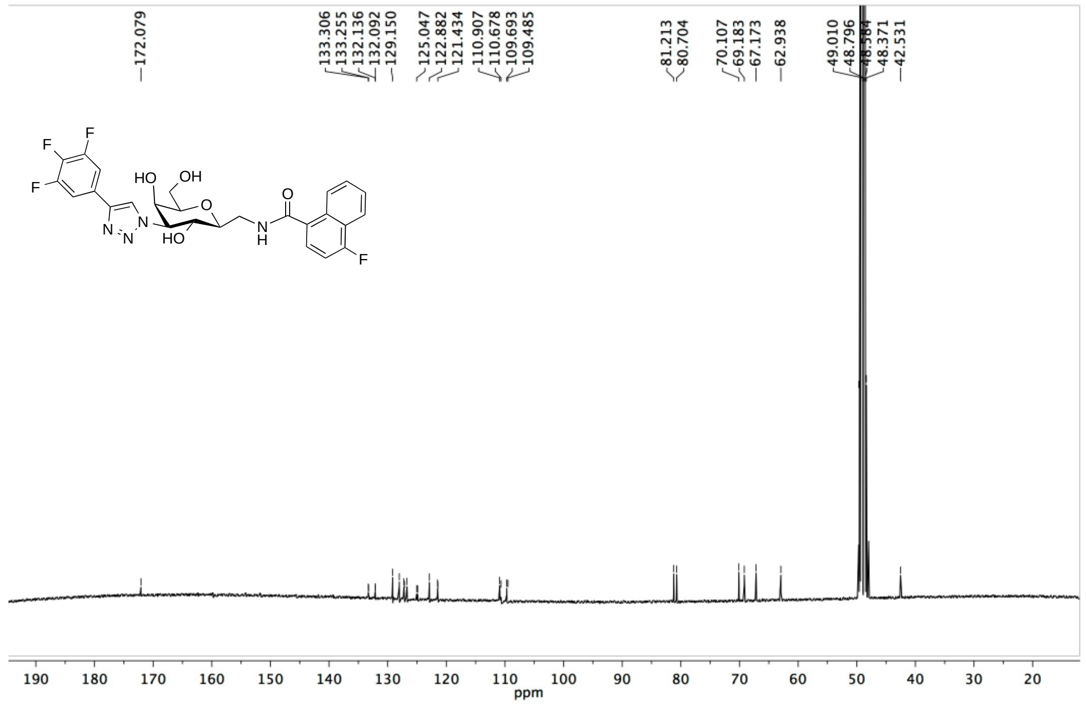

Supplement: Supplementary file 1 [file molecules-24-04554-s001.pdf]
